# Cost-effectiveness analysis of molecular testing in minimally invasive samples to detect endometrial cancer in women with postmenopausal bleeding

**DOI:** 10.1038/s41416-023-02291-1

**Published:** 2023-05-10

**Authors:** Paula Peremiquel-Trillas, David Gómez, José Manuel Martínez, Sergi Fernández-González, Jon Frias-Gomez, Sonia Paytubi, Beatriz Pelegrina, Marta Pineda, Joan Brunet, Jordi Ponce, Xavier Matias-Guiu, Xavier Bosch, Silvia de Sanjosé, Laia Bruni, Laia Alemany, Laura Costas, Mireia Díaz

**Affiliations:** 1 Cancer Epidemiology Research Programme, Catalan Institute of Oncology. Av Gran Vía 199-203, 08908L’Hospitalet de Llobregat, Barcelona, Spain; 2 Bellvitge Biomedical Research Institute—IDIBELL. Av Gran Vía 199-203, 08908L’Hospitalet de Llobregat, Barcelona, Spain; 3 Faculty of Medicine, University of Barcelona. C/ Casanova, 143, 08036 Barcelona, Spain; 4 Consortium for Biomedical Research in Epidemiology and Public Health—CIBERESP. Carlos III Institute of Health. Av. De Monforte de Lemos 5, 28029 Madrid, Spain; 5 Department of Gynecology and Obstetrics, Hospital Universitari de Bellvitge, IDIBELL. Hospitalet de Llobregat, Barcelona, Spain; 6 Hereditary Cancer Program, IDIBELL. Catalan Institute of Oncology. Hospitalet de Llobregat, Barcelona, Spain; 7 Consortium for Biomedical Research in Cancer—CIBERONC. Carlos III Institute of Health. Av. De Monforte de Lemos 5, 28029 Madrid, Spain; 8 Medical Oncology Department. Catalan Institute of Oncology, Doctor Josep Trueta Girona University Hospital. Av. França-Sant Ponç s/n, 17007 Girona, Spain; 9 Department of Pathology, Hospital Universitari de Bellvitge, IDIBELL. Hospitalet de Llobregat, Barcelona, Spain; 10 Faculty of Health Sciences, UOC - Open University of Barcelona, Barcelona, Spain; 11 ISGlobal, Barcelona, Spain; 12 Consultant National Cancer Institute, National Institutes of Health, Bethesda, Maryland USA

**Keywords:** Translational research, Experimental models of disease

## Abstract

**Introduction:**

New approaches are being developed to early detect endometrial cancer using molecular biomarkers. These approaches offer high sensitivities and specificities, representing a promising horizon to develop early detection strategies.

**Objective:**

To evaluate the effectiveness and cost-effectiveness of introducing molecular testing to detect endometrial cancer in women with postmenopausal bleeding compared to the current strategy using the national healthcare service perspective.

**Methods:**

A Markov model was developed to assess the two early detection strategies. The model predicts the number of hysterectomies, lifetime expectancy, quality-adjusted life-years, endometrial cancer prevalence and incidence, mortality from endometrial cancer and the lifetime cost of screening, diagnosis, and treatment. Strategies were compared using the incremental cost-effectiveness ratio.

**Results:**

The molecular strategy reduces 1.9% of the overall number of hysterectomies and the number of undetected cancer cases by 65%. Assuming a molecular test cost of 310€, the molecular strategy has an incremental cost of -32,952€ per QALY gained, being more effective and less expensive than the current strategy.

**Conclusions:**

The introduction of molecular testing to diagnose endometrial cancer in women presenting postmenopausal bleeding provides more health benefit at a lower cost, and therefore has the potential to be cost-effective.

## Introduction

Endometrial cancer (EC) is the sixth most common cancer in women worldwide. Its incidence is on rise due to an increased prevalence of metabolic syndrome and obesity, as well as population ageing [1]. Abnormal uterine bleeding occurs in 90% of women with EC, but only 9% of women with postmenopausal bleeding (PMB) will be diagnosed with EC [2]. All abnormal uterine bleeding requires further evaluation to identify and treat a potentially severe condition [3, 4].

The usual standard of care strategy to diagnose EC consists of transvaginal ultrasound (TVU) performance among symptomatic women and endometrial sampling in cases of increased endometrial thickness [5, 6]. EC diagnosis requires histological diagnosis of endometrial samples obtained in gynaecological outpatient visits. A biopsy with pipelle has been the election method to obtain these samples, overcoming dilation and curettage limitations [7–10]. Nevertheless, one of the main inconveniences of pipelle is its failure rate (due to scarce material or cervical stenosis) and the possibility of obtaining false negative results due to blind sampling [11, 12].

Nowadays, new molecular approaches are being developed to early detect EC using genomics, epigenomics and proteomics in endometrial samples and cervicovaginal samples [3, 13–17]. These new approaches benefit from the anatomical continuity of the uterine cavity with the cervix [13–15]. The molecular characterisation of detached cells permits the differentiation between EC patients and healthy women with high sensitivity and specificity [8, 14, 15, 18]. Molecular tests can be sensitive with less amount of material, and they could be particularly beneficial in cases of sampling insufficiency. Also, they could be better tolerated if performed on non-invasive samples. These advances offer a promising new horizon to develop early detection strategies, especially among symptomatic women and high-risk populations.

The implementation of novel diagnostic approaches is usually associated with increased costs, and budgetary constraints in different settings could mean that not all available early detection strategies may be included in the healthcare system. In this context, Markov simulation models are frequently used to model the underlying disease process, evaluate the potential long-term effectiveness and costs of alternative strategies. These models offer a coherent approach for integrating clinical research evidence and patient values to optimise choices and maximise population health benefits. Thus, these models could be useful to assess whether the health benefits of novel interventions overcome their costs in order to inform health planners and decision-makers about the best possible allocation and most efficient use of healthcare resources.

The present study aims to evaluate the effectiveness and cost-effectiveness of introducing molecular testing in minimally invasive samples to detect EC in women with postmenopausal bleeding compared to the usual standard of care diagnostic strategy.

## Methods

More detailed information on the simulation model and cost-effectiveness analysis can be found in Supplementary Appendix.

### Base model

A time-independent Markov model was developed to assess the effectiveness and cost-effectiveness of two early detection strategies for EC (Supplementary Appendix). The model consists of 6 mutually exclusive and collectively exhaustive health states: PMB, Detected EC, No detected EC, EC survivor, Death from EC, and Death from other causes. All simulated women are assumed to be healthy at baseline in terms of EC, but all of them are menopausal women with recurrent uterine bleeding during the simulated time horizon. Since it is an initial condition and not a symptom that can occur during the simulation, it has been considered as the initial health state. Thus, this closed model follows a single cohort of 50-year-old women with PMB (considered as the average age of menopause) who can move from one health state to another according to some transition probabilities using 1-year increments until age 85 years (time horizon of 35 years) or die. Women may die from EC in the cancer stage or from other causes in every health state and every cycle. The model was coded in R [19]. Further details are provided in Supplementary Appendix.

### Strategies considered

**Fig. 1:**
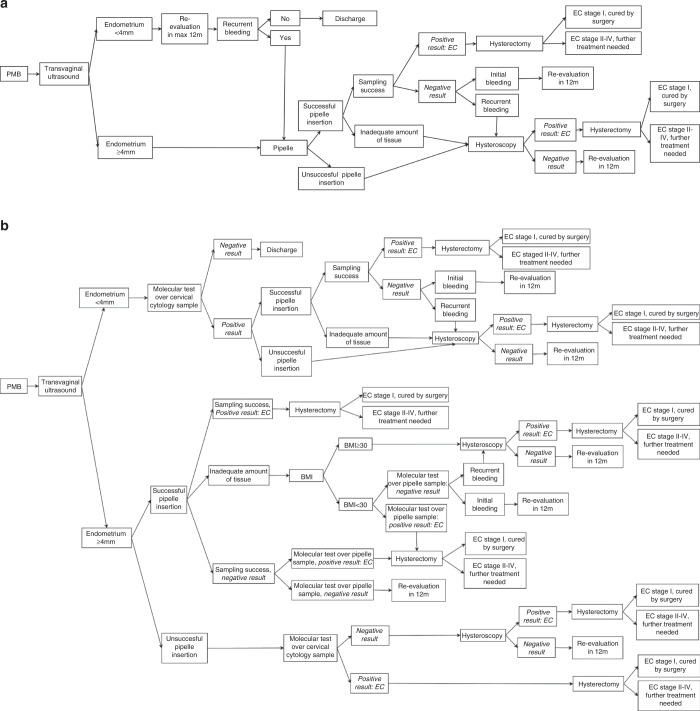
Algorithms for assessing postmenopausal bleeding. **a** Current strategy (regular practice). **b** Proposed molecular strategy including molecular diagnosis. BMI body mass index, EC endometrial cancer, PMB postmenopausal bleeding. **a** The current regular practice consists in the performance of a transvaginal ultrasound at the first gynaecological consultation followed by pipelle endometrial biopsy when an increased endometrial thickness is detected in the transvaginal ultrasound. **b** The proposed molecular strategy for PMB assessment includes molecular testing on minimally invasive samples (cervicovaginal pap brush sample) and pipelle endometrial biopsy.

The usual practice for PMB assessment (Fig. 1a) consists of a TVU at the first gynaecological visit followed by pipelle biopsy in cases of increased endometrial thickness. Hysteroscopy is performed if pipelle insertion is the unsuccessful insertion or scarce material is obtained. If endometrial biopsy indicates the presence of neoplastic or preneoplastic disease, a hysterectomy is performed. This strategy is compared with a proposed hypothetical novel molecular strategy for PMB assessment (Fig. 1b). In summary, a molecular test is proposed in a cervical Pap brush sample if the vaginal ultrasound is negative or the pipelle insertion is unsuccessful, as well as a molecular test in endometrial samples when the corresponding morphologic results are negative, or the obtained amount is insufficient for a morphologic diagnosis. The test performance of this hypothetical molecular test is assumed to be similar to those of other molecular test published [13, 14, 20].

### Health input parameters

Time-independent input values were based on the best-available data from an extensive literature search regardless of a specific setting as we assume the mechanism of endometrial carcinogenesis does not fundamentally differ between countries (Table 1 and Supplementary Appendix). However, as epidemiologic burden differs between geographic regions due to a different distribution of risk factors, we selected age-specific EC prevalence and mortality from Spain. Further details on health input parameters are detailed in Supplementary Appendix.

**Table 1:** Summary of base-case values, ranges, and data sources for model input parameters.

Parameters	Input value	Range for sensitivity analysis	Source
Prevalence			
Prevalence of EC in women aged 50 + (per 100,000)	Age-dependent (overall value 53.5)^*^	Age-dependent^**^	Globocan [38]
Prevalence of EC in women with PMB (%)	9.0	8.0–11.0	Clarke et al. [2]
Prevalence of PMB in women with EC	0.91	0.87–0.93	Clarke et al. [2]
Proportion of undetected EC cases progressing in 1 year			
Progressing from Stage I to II	0.30	0.285–0.315^**^	Expert consensus, Chen et al. [39]
Progressing from Stage II to III	0.40	0.38–0.42^**^	Expert consensus, Chen et al. [39]
Progressing from Stage III to IV	0.55	0.52–0.578^**^	Expert consensus, Chen et al. [39]
Proportion of EC cases (50 + years)			
Stage I	Age-dependent (overall value 0.705)^*^	Age-dependent^**^	SEER 18 [40] (white females aged 50+ years)
Stage II	Age-dependent (overall value 0.062)^*^	Age-dependent^**^	SEER 18 [40] (white females aged 50+ years)
Stage III	Aage-dependent (overall value 0.149)^*^	Age-dependent^**^	SEER 18 [40] (white females aged 50+ years)
Stage IV	Age-dependent (overall value 0.084)^*^	Age-dependent^**^	SEER 18 [40] (white females aged 50+ years)
Sensitivities, specificities, and probabilities of success			
Sensitivity of hysteroscopy (PMB)	0.864	0.821–0.907^**^	Cooper et al. [4]
Specificity of hysteroscopy (PMB)	0.992	0.942–1.0^**^	Clark et al. [41]
Sensitivity of Pipelle (PMB)	0.94	0.84–0.99	Yi et al. [11]
Specificity of Pipelle (PMB)	0.99	0.98–1.0	Yi et al. [11]
Sensitivity of TVU (4 mm)	0.906	0.861–0.951^**^	Patel et al. [6]
Specificity of TVU (4 mm)	0.235	0.223–0.247^**^	Patel et al. [6]
Sensitivity of molecular test (Pap brush sample)	0.78	0.75–0.85	Reijnen et al. [42], Wang et al. [14]
Specificity of molecular test (Pap brush sample)	0.97	0.83–1.0	Reijnen et al. [42]
Sensitivity of molecular test (Pipelle)	0.96	0.92–0.98	Reijnen et al. [42]
Specificity of molecular test (Pipelle)	0.94	0.79–0.99	Reijnen et al. [42]
Obesity			
Proportion of obese women aged 55-64 (BMI > 30)	Age-dependent (overall value 0.213)^*^	Age-dependent^**^	Eurostat 2014 (Spanish women)
HR of EC for BMI > 30 versus BMI 22	3.0	2.5–4.0	Bhaskaran et al. [43]
Other probabilities			
Probability of bleeding recurrence	0.80	0.76–0.84^**^	Expert consensus
Probability of Pipelle success (insertion)	0.92	0.89–0.94	Clark et al. [12]
Probability of Pipelle success (tissue)	0.87	0.86–0.90	Clark et al. [12]
Recurrence rate			
EC Stage I	0.065	0.061–0.067^**^	Huijgens et al. [44]
EC Stage II	0.20	0.19–0.21^**^	Huijgens et al. [44]
EC Stage III	0.375	0.356–0.394^**^	Huijgens et al. [44]
EC Stage IV	0.667	0.634–0.7^**^	Huijgens et al. [44]
5-year survival rates and probability of death			
EC Stage I	Age-dependent (overall value 0.957)^*^	Age-dependent^**^	SEER 18 [40] (white females aged 50+ years)
EC Stage II	Age-dependent (overall value 0.707)^*^	Age-dependent^**^	SEER 18 [40] (white females aged 50+ years)
EC Stage III	Age-dependent (overall value 0.707)^*^	Aage-dependent^**^	SEER 18 [40] (white females aged 50+ years)
EC Stage IV	Age-dependent (overall value 0.171)^*^	Age-dependent^**^	SEER 18 [40] (white females aged 50+ years)
Probability of death from other causes	Age-dependent (overall value 0.017)^*^	Aage-dependent^**^	INE [45], Globocan [38]
Utilities			
Bleeding	0.95	0.903–0.998^**^	Lete et al. [21]
Non-EC hysterectomy	0.70	0.665–0.735^**^	Expert consensus
EC Stage I	0.68	0.646–0.714^**^	Goldie et al. [23], Kwon et al. [46]
EC Stage II	0.56	0.532–0.588^**^	Goldie et al. [23]
EC Stage III	0.56	0.532–0.588^**^	Goldie et al. [23]
EC Stage IV	0.48	0.456–0.504^**^	Goldie et al. [23]
Undetected EC	0.68	0.646–0.714^**^	Expert consensus
Survival Stage I	0.88	0.834–0.922^**^	Goldie et al. [23], Kwon et al. [46]
Survival Stage II	0.72	0.687–0.760^**^	Goldie et al. [23], Kwon et al. [46]
Survival Stage III	0.72	0.687–0.760^**^	Goldie et al. [23]
Survival Stage IV	0.62	0.589–0.651^**^	Goldie et al. [23]
Costs (expressed in €2020)			
Initial visit	148	140.6–155.4^**^	DOGC [47]
Successive visit	69	65.5–72.5^**^	DOGC [47]
Telephone visit	41	38.9–43.1^**^	DOGC [47]
Pap smear^a^	49	46.6–51.5^**^	DOGC [47]
TVU	31	29.5–32.6^**^	DOGC [47]
Pipelle^a^	105	99.8–110.3^**^	DOGC [47]
Molecular test	310	294.5–325.5^**^	DOGC [47]
Hysteroscopy	134	127.3–140.7^**^	DOGC [47]
Hysterectomy (Stage I)^b^	5367	3817–6917	DOGC [47]
Hysterectomy (Stages II–IV)^b^	10,607	10,077–11,137^**^	DOGC [47]
EC adjuvant treatment^c^	4326	3743–8653	DOGC [47]

*EC* endometrial cancer, *PMB* postmenopausal bleeding, *BMI* body mass index, *HR* hazard ratio, *TVU* transvaginal ultrasound.

*Age-dependent values were used in the model (See supplementary Appendix). We included the overall value in the table as a reference.

**As the range is not available from scientific literature, a ± 5% was assumed of the base value.

^a^Includes sample obtention, device and pathological techniques.

^b^Includes perioperative management according to FIGO stage: a full blood count and liver and renal function profiles, pelvic MRI, as well as additional imaging tests (CT scan and/or FDG-PET-CT) in those patients at higher risk of extrapelvic disease.

^c^Includes chemotherapy and/or radiotherapy and/or brachytherapy.

### Utilities

Utility scores reflect the health status or outcome of a patient, ranging from 0 (reflecting death) to 1 (reflecting perfect health). Utilities remain fixed over time. Each health state or condition is assigned a utility and the contribution of this utility depends on the length of time spent in the state. Utilities for bleeding and EC Stages I–II–III–IV were extracted from the literature (Table 1 and Supplementary Appendix) [21–23]. Women with EC spend only one year in the detected EC state because they are assumed to be cured after treatment. Therefore, utilities by stage are applied only once. In the next cycle, women go into the EC survivor state which has also associated utilities depending on the stage. In subsequent cycles, women may develop recurrence and return to the detected EC state. Hysterectomised women with no EC have an initial utility of 0.95 as bleeding women, but as there is a temporary quality of life decrement related to surgery and moderate to severe pain, we assumed a final utility of 0.7 [24]. The utility for women with undetected EC was assumed as the utility of EC Stage I. Further details on utilities are detailed in Supplementary Appendix.

### Cost data

The analysis was performed by the national healthcare system perspective including only individual patient healthcare costs of screening, diagnosis, and treatment (Table 1) [25]. Further details on cost data are provided in Supplementary Appendix.

### Outcomes, measurements, and cost-effectiveness analysis

For each strategy, the model predicts the number of hysterectomies, lifetime expectancy, quality-adjusted life-years (QALYs), EC prevalence and incidence, mortality from EC and the lifetime cost of screening, diagnosis, and treatment. The QALY combines into a single index the length of life (life expectancy) and the quality of life (utility score) multiplying the utility value of a given health state by the years lived in that state (e.g., one QALY is equal to 1 year of life in perfect health) [26, 27]. The incremental cost-effectiveness ratio (ICER) is the measurement used in cost-effectiveness analysis expressed as the ratio of the difference in cost (euros) between the two strategies to the difference in health (QALYs). Therefore, it represents the incremental cost associated with one additional QALY gained. The cost-effectiveness threshold which is the maximum amount a decision-maker is willing to pay for a unit of health outcome (in our case, QALY) has stated to be between 22,000–25,000€ in Spain [28, 29]. Strategies associated with an ICER below this threshold will be judged as cost-effective. Both cost and health outcomes were discounted at an annual rate of 3% as the present value of money or health is viewed as higher than the expected value of heath and financial returns in the future [30].

### Sensitivity analysis

Deterministic and probabilistic sensitivity analyses were carried out for the most influential parameters to determine the robustness of the results and identify key parameters that could drive results. One-way deterministic sensitivity analysis (DSA) varies the value of one specific parameter whilst holding all other parameters fixed to assess its isolated effect on the results. Values were varied in plausible ranges based on published data and expert opinion, but for those with unknown ranges, were varied by ±5% of the base value.

In probabilistic sensitivity analysis (PSA), a distribution is assigned to each parameter, and the ranges are determined by the mean value, the standard deviation, and the shape of the data [31]. Probabilities and utilities were modelled using beta distributions, while gamma distributions were used for costs [32]. The PSA was conducted with 1000 Monte–Carlo iterations. Further details on sensitivity analysis can be found in Supplementary Appendix.

### Ethical aspects

The research did not meet the criteria for human subjects research as data was obtained from a published literature review, thus informed consent was not required.

## Results

### Health results

**Fig. 2:**
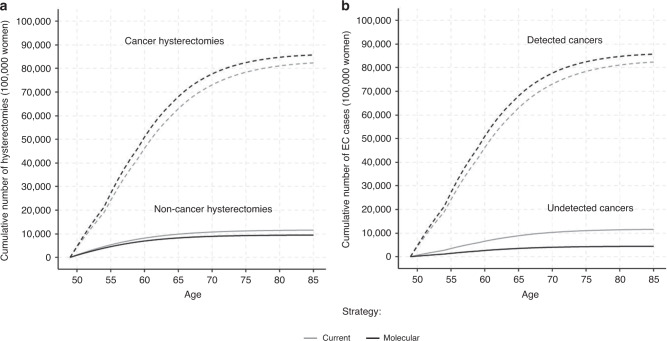
Summary of health results. **a** Cumulative number of non-cancer and cancer hysterectomies performed. **b** Detected and undetected cancers by age.

The mean annual number of hysterectomies and cancers detected and undetected by age groups for the two strategies and the lifetime cumulative numbers by age groups are presented in Fig. 2 and Supplementary Appendix. The molecular strategy yields a decrease of 1.9% of the overall number of hysterectomies compared to the usual strategy. However, there is an increase in the number of hysterectomies among women with EC (0.9%) that it is only observed in younger groups (8.5%, 5.3% and 0.5% in the 50–54, 55–59 and 60–64 age groups, respectively) as there is a decrease among older women (−5.3% and −14.5% in 65–69 and 70+ age groups, respectively). The molecular strategy results in an average of 20.5% fewer hysterectomies in women with no EC compared to the usual strategy, and this decrease in hysterectomies is higher at older ages (−16.3% and −34.0% in 50–54 and 70+ age groups, respectively). The total number of undetected EC decreases by 65% in the molecular strategy compared to the usual strategy (4.8% versus 12.8%, respectively).

### Cost-effectiveness and sensitivity analyses

Assuming a cost of 310€ per molecular test, the results of the cost-effectiveness analysis for the base-case scenario show that the strategy including molecular diagnosis has an incremental cost of -32,952€ per QALY gained compared with the usual strategy. This cost is not only below the usual cost-effectiveness threshold in Spain (€22,000 - €25,000 per QALY), but is also negative, which in this case means is both more effective and less expensive than the usual strategy (Supplementary Appendix).

The one-way DSA for the parameters with known ranges shows that the strategy including molecular markers remains cost-saving except when the specificity of the molecular test on cervicovaginal Pap brush samples is reduced to 0.83. In this case, there is no increase in terms of the effectiveness of the molecular strategy, but it is still less costly than the usual strategy, and the ICER remains below the cost-effectiveness threshold (Supplementary Appendix). In this case, the implementation of the strategy with the molecular test would cost €13,376 per QALY gained. To exclude the effect of utilities and to check for possible differences in study findings, results in life-years gained (LYG) are also reported (Supplementary Appendix), although no discrepancies are observed.

**Fig. 3:**
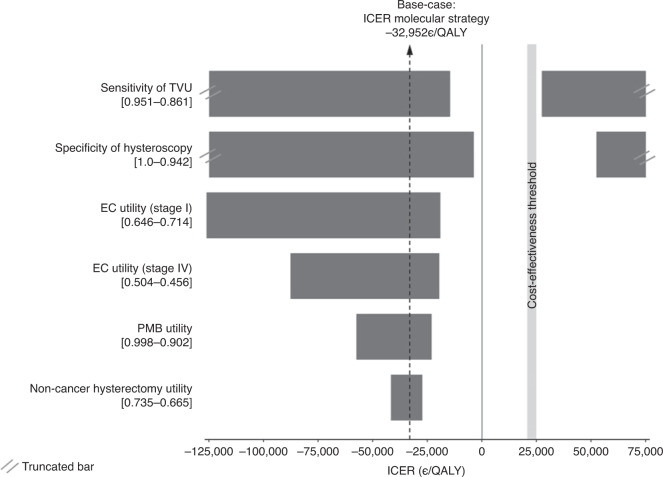
One-way deterministic sensitivity analysis for selected parameters with unknown ranges. ICER incremental cost-effectiveness ratio, PMB postmenopausal bleeding, QALY quality-adjusted life-years, TVU transvaginal ultrasound. One-way sensitivity analysis depicted in a tornado diagram. The *y* axis lists the parameters that are modified in the analysis. The *x* axis shows the effect on the ICER of the modification of those parameters. The bars indicate the change in the ICER caused by changes in the value of the indicated variable holding all other parameters similar. The values in brackets indicate the range of the parameter. The cost-effectiveness threshold is set at 22,000–25,000 €/QALY.

The one-way DSA for the parameters with unknown ranges (Fig. 3 shows the most variable parameters and Supplementary Appendix the complete DSA) shows that the cost-effectiveness of the proposed strategy including molecular markers remains relatively stable. However, the results are sensitive to some parameters, especially to a decrease in the TVU sensitivity and hysteroscopy specificity where the molecular strategy could not always be cost-effective. The results are also sensitive to some utilities which in some cases would make the molecular strategy less effective but ICERs will still remain below the cost-effectiveness threshold.

The PSA to assess the impact of the molecular test cost in the results is shown in the Supplementary Appendix. When the molecular test cost is set at 310€, 92.6% of the simulations remain cost-effective. Assuming twice the cost of the baseline scenario, the probability of cost-effectiveness would decrease by almost half. The probability for the molecular strategy to be cost-effective at different WTP values and molecular cost test is shown in the Supplementary Appendix.

**Fig. 4:**
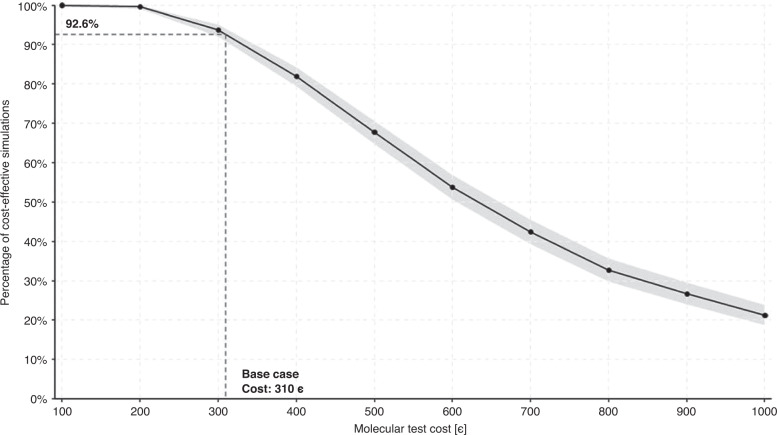
Probabilistic sensitivity analysis for the cost of the molecular test. Percentage of cost-effective simulations for the molecular strategy according to different molecular test values (black line). The *y* axis represents the cost-effectiveness probability of the molecular strategy simulations. The *x* axis represents the cost in € of the molecular test. The grey shadow represents the 95% CI.

In order to identify the importance of each individual parameter, a univariate probabilistic approach was first performed (Supplementary Appendix). Subsequently, a multivariate PSA was performed that simultaneously modified parameters with the greatest impact on the model (TVU sensitivity, hysteroscopy specificity, utilities for Stage I and IV EC and PMB utilities) (Fig. 4). Cost-effectiveness planes show that 63.7% of the simulations were below the cost-effectiveness threshold when deviations were set at a tenth the base value and decreased to 51.2% when deviations were increased at a sixth of the base value. Assuming the low deviation (a tenth of the base value), the probability of the usual strategy would never exceed 50% with a cost-effectiveness limit range of €50,000/QALY. The corresponding curve with a higher deviation (a sixth of the base value) shows that at a threshold ratio lower than €30,000/QALY, the molecular strategy becomes the preferred strategy, with a higher probability of being cost-effective (Supplementary Appendix).

## Discussion

To our knowledge, this is the first study presenting an economic evaluation of a strategy to introduce molecular testing for EC diagnosis in endometrial and cervicovaginal samples among women presenting PMB. Our study suggests that the diagnosis strategy to assess PMB including molecular testing is very cost-saving compared to the usual diagnostic strategy for a given cost of 310€ per molecular test. Although sensitivity analyses show that outcomes are relatively stable, the results are sensitive to some parameters, especially to a decrease in the TVU sensitivity and hysteroscopy specificity.

To implement novel early detection and screening approaches in clinical settings, the evaluation of effectiveness and cost-effectiveness is fundamental to allocate resources efficiently. So far, only a few studies have performed economic evaluations of different strategies to evaluate EC [33]. Of these, only one has included women presenting PMB. Yi et al. [11] compared the performance of two different endometrial sampling techniques to detect EC among PMB, being pipelle use the most cost-effective strategy. Havrilesky et al. [34] evaluated the cost-effectivity of introducing a high accurate serum biomarker (98% sensitivity, 98% specificity) as an annual screening test to detect EC in the general population compared to no screening, annual endometrial biopsy and annual TVU. Their results showed that using this biomarker is potentially cost-effective among obese women and tamoxifen users. Although PMB women were not considered, these results also suggest that accurate novel biomarkers in minimally invasive samples could be considered good value for money in high-risk populations overcoming the actual available diagnostic possibilities. Recently, Warring et al. [35] evaluated the cost of EC diagnose in women presenting abnormal uterine bleeding and concluded that the current available procedures yield substantial costs even when a benign diagnosis was obtained.

Studies evaluating the accuracy of molecular testing over gynaecological samples have obtained higher performance than currently available histopathological techniques [13–15, 20]. Costas et al. [15] concluded that those novel techniques offer a promising perspective for EC diagnosis as well as prevention, due to their high accuracy. In particular, Wang et al. [14] showed a sensitivity of 81% and a specificity of 99% with the PapSEEK molecular test using Pap brush samples among 382 EC patients. Similarly, Reijnen et al. [13] obtained 96%/78%, sensitivities and 94%/97% specificities in pipelle and cervical samples, respectively, when they evaluated 59 EC cases and 31 controls using a 8-genes next-generation sequencing panel. Our study shows that the introduction of molecular markers for the diagnosis of EC in endometrial and cervical samples yields a decrease of 1.9% of the overall number of hysterectomies compared to the usual strategy. Nevertheless, we observe an increase in the overall number of hysterectomies among women with EC (0.9%), only observed in women ≤65 years of age, suggesting that we could detect EC earlier with molecular tests. Also, the molecular strategy yields in an average reduction of 20.5% of hysterectomies in women with no EC, which is observed consistently among all age groups. Importantly, the number of undetected cancers decreases by 65% with the molecular strategy.

Developing models there will always be a trade-off, both in the selected approach and in the construction of the case study in which certain details will be excluded. Markov cohort models are a common and exhaustive approach to perform cost-effectivity evaluations, and they are suitable for modelling conditions whose events occur repeatedly over time. However, unlike individual-based models that keep track of each individual’s path and can accommodate more complex strategies, cohort models operate at an aggregate level by limiting the number of health states and the detail of the strategies. This, however, favours Markov models being more transparent, relatively straightforward to develop, analyse, and communicate, provided the number of health states is not excessive [36]. The cohort simulated in this study constitutes a closed cohort of PMB women; therefore, no other women enter the cohort, and women are considered to remain with PMB during the whole simulation. The main limitation of Markov models is the memoryless property, whereby the probability of making the transition from one state to another is independent of the path taken to reach that state; that is, transition probabilities depend neither on past states nor on the time spent in the current [37]. The implication is that homogeneity is assumed within health states since all those within a given health state will have the same probability of making the transition to any other state. In addition, although a common technique, the probability of annual EC death and survival for all cycles is calculated with an exponential distribution using survival rates. Therefore, if the distribution deviates from the current mortality data, the number of deaths estimated from EC could differ. As with all simulation models, the results of our study rely on the quality of the data used for the input parameters. Although estimates have been obtained from an extensive literature review, and the most uncertain values have been discussed with a panel of experts, drastic changes in the values of these parameters or assumptions would require a review of the analyzes. Although most EC patients present abnormal uterine bleeding (90%), women without bleeding have not been considered in the model. Also, we have been conservative in the construction of the molecular algorithm, maintaining the actual diagnostic steps. Probably, once molecular tests for EC diagnosis are fully validated and available in the clinical setting, the algorithm could be simplified. As new data emerges on the recent molecular classification of EC, it will be relevant to modify the proposed model and integrate the latest data available to provide more accurate guidance for future policy and clinical practice. Additionally, simulating the cost-effectiveness of this novel tests according to the different EC subtypes would be valuable, particularly for those more aggressive subtypes of EC, where molecular tests for EC detection could be particularly beneficial. Further work is warranted to assess the potential benefit of the introduction of molecular tests for EC screening purposes among asymptomatic population. We should note that our model, which is focused on the use of molecular tests for diagnostic purposes in symptomatic patients (PMB), does not consider biases related to screening, such as overdiagnosis or lead-time bias. Overdiagnosis in cancer screening may result in unnecessary treatment of low-risk patients, and thereby increase costs and inflate survival, while lead-time bias may prolong the time between diagnosis and death, potentially reducing costs and inflate survival. However, neither case affects our model. Survival is an input of the model based on the SEER survival which does not include population with EC screening. The number of cases to which we apply the costs is also an input of the model extracted from the prevalence of EC in Spain in the general population where no screening is done and the risk of this cancer in PMB who are symptomatic women. Also, since we use a time-independent Markov model, the diagnostic advance data in the cohort is not affected. It is uncertain how these two outcomes could offset each other in our model, and it will be crucial to consider their impact on the results in a screening setting.

## Conclusion

Our results suggest that the introduction of molecular testing in endometrial and cervical specimens for EC diagnosis among women presenting PMB has the potential to be cost-effective. Continuous advances are being made to develop molecular tests that can be ultimately applied to clinical practice. The current analysis should be updated once new results and novel tests are validated and commercialised. Other industrialised countries with a similar EC burden and health infrastructures may also benefit from these results. Our findings provide valuable information for health decision-makers to guide resource allocation decisions, to inform future policy and to facilitate more effective and efficient diagnosis algorithms for EC diagnosis. Nevertheless, before its implementation, it is essential to perform a budgetary impact analysis of their introduction into the healthcare system.

## Supplementary information

Supplementary Appendix Final version

## Data Availability

Data are available upon request to the corresponding author.
