# Supplementary material for: Cost-effectiveness analysis of molecular testing in minimally invasive samples to detect endometrial cancer in women with postmenopausal bleeding
Source: Br J Cancer. 2023 May 10;129(2):325–34. doi: 10.1038/s41416-023-02291-1 (PMC10338433; doi:10.1038/s41416-023-02291-1)
Supplement: Supplementary file 1 — Supplementary Appendix Final version [file 41416_2023_2291_MOESM1_ESM.docx]

**SUPPLEMENTARY APPENDIX**

**Accompanying the manuscript:**

**Cost-effectiveness analysis of molecular testing in minimally invasive samples to detect endometrial cancer in women with postmenopausal bleeding**

Methods 3

Table S1. Consolidated Health Economic Evaluation Reporting Standards (CHEERS) checklist 2022 3

Table S2. Summary of key design criteria for the model simulation and cost-effectiveness analysis. 5

**Model** 5

Figure S1. State transition diagram of the Markov Model. 6

Input values, parameters, and assumptions 7

**Health input data** 7

Table S3. Proportion of cancer stage at diagnosis according to age groups. 8

Table S4. Summary of base case values according to age-group for model input parameters. 9

Table S5. Mortality rates and probabilities according to age groups. 10

**Utilities** 10

**Cost input data** 10

Calibration 11

Figure S2. Theorical prevalence data and uncalibrated model-predicted values. 12

Figure S3. Theorical prevalence data and calibrated model-predicted values. 12

Sensitivity analysis 13

Results 14

Figure S4: Annual number of hysterectomies and cancers by age group (A-E) and overall (F) 14

Table S6. Mean annual number of hysterectomies and cancers by age groups and overall. 15

**Sensitivity Analysis** 15

Table S7. One-way deterministic sensitivity analysis of parameters with known ranges. Cost-effectiveness in € per QALY gained. 16

Table S8. One-way deterministic sensitivity analysis of parameters with known ranges. Cost-effectiveness in € per LY gained. 18

Figure S5. One-way deterministic sensitivity analysis for selected parameters with unknown ranges. Parameter values were varied by ±5% of the base value. 20

Figure S6. Cost-effectiveness acceptability at different willingness to pay values and costs of the molecular test. 21

Figure S7. Incremental cost-effectiveness plane for parameters with known ranges. 22

Figures S8-S57. Univariate probability sensitivity analysis (PSA) for all parameters using as standard deviation (SD) a tenth of the base case value (SD= base value/10). 1

Figure S58. Multivariate probabilistic sensitivity analysis including bleeding persistence, sensitivity of TVU, specificity of TVU, utilities of EC stage I and stage IV. 7

# Methods

This cost-effectiveness analysis follows the Consolidated Health Economic Evaluation Reporting Standards (CHEERS) statement for the reporting standards of a health intervention economic evaluation^1^.

### Table S1. Consolidated Health Economic Evaluation Reporting Standards (CHEERS) checklist 2022

| **Topic** | **No.** | **Item** | **Location where item is reported** |
| --- | --- | --- | --- |
| **Title** |  |  |  |
|  | 1 | Identify the study as an economic evaluation and specify the interventions being compared. | Page 1 |
| **Abstract** |  |  |  |
|  | 2 | Provide a structured summary that highlights context, key methods, results, and alternative analyses. | Page 4 |
| **Introduction** |  |  |  |
| **Background and objectives** | 3 | Give the context for the study, the study question, and its practical relevance for decision making in policy or practice. | Page 5-6 |
| **Methods** |  |  |  |
| **Health economic analysis plan** | 4 | Indicate whether a health economic analysis plan was developed and where available. | Page 6-8 and Appendix |
| **Study population** | 5 | Describe characteristics of the study population (such as age range, demographics, socioeconomic, or clinical characteristics). | Page 6-8 and Appendix |
| **Setting and location** | 6 | Provide relevant contextual information that may influence findings. | Page 76-8 and Appendix |
| **Comparators** | 7 | Describe the interventions or strategies being compared and why chosen. | Page 6-8, Figure 1 and Appendix |
| **Perspective** | 8 | State the perspective(s) adopted by the study and why chosen. | Page 6-8 and Appendix |
| **Time horizon** | 9 | State the time horizon for the study and why appropriate. | Page 6-8 and Appendix |
| **Discount rate** | 10 | Report the discount rate(s) and reason chosen. | Page 6-8 and Appendix |
| **Selection of outcomes** | 11 | Describe what outcomes were used as the measure(s) of benefit(s) and harm(s). | Page 6-8 and Appendix |
| **Measurement of outcomes** | 12 | Describe how outcomes used to capture benefit(s) and harm(s) were measured. | Page 6-8 and Appendix |
| **Valuation of outcomes** | 13 | Describe the population and methods used to measure and value outcomes. | Page 6-8 and Appendix |
| **Measurement and valuation of resources and costs** | 14 | Describe how costs were valued. | Page 6-8 and Appendix |
| **Currency, price date, and conversion** | 15 | Report the dates of the estimated resource quantities and unit costs, plus the currency and year of conversion. | Page 6-8 and Appendix |
| **Rationale and description of model** | 16 | If modelling is used, describe in detail and why used. Report if the model is publicly available and where it can be accessed. | Page 6-8, Figure 1 and Appendix |
| **Analytics and assumptions** | 17 | Describe any methods for analysing or statistically transforming data, any extrapolation methods, and approaches for validating any model used. | Page 6-8 and Appendix |
| **Characterising heterogeneity** | 18 | Describe any methods used for estimating how the results of the study vary for subgroups. | Page 6-8 and Appendix |
| **Characterising distributional effects** | 19 | Describe how impacts are distributed across different individuals or adjustments made to reflect priority populations. | Page 6-8and Appendix |
| **Characterising uncertainty** | 20 | Describe methods to characterise any sources of uncertainty in the analysis. | Page 6-8 and Appendix |
| **Approach to engagement with patients and others affected by the study** | 21 | Describe any approaches to engage patients or service recipients, the general public, communities, or stakeholders (such as clinicians or payers) in the design of the study. | Not Applicable |
| **Results** |  |  |  |
| **Study parameters** | 22 | Report all analytic inputs (such as values, ranges, references) including uncertainty or distributional assumptions. | Page 9-10, Figure 2&3 and Appendix |
| **Summary of main results** | 23 | Report the mean values for the main categories of costs and outcomes of interest and summarise them in the most appropriate overall measure. | Page 9-10, Figure 2&3 and Appendix |
| **Effect of uncertainty** | 24 | Describe how uncertainty about analytic judgments, inputs, or projections affect findings. Report the effect of choice of discount rate and time horizon, if applicable. | Page 9-10, Figure 2&3 and Appendix |
| **Effect of engagement with patients and others affected by the study** | 25 | Report on any difference patient/service recipient, general public, community, or stakeholder involvement made to the approach or findings of the study | Not Applicable |
| **Discussion** |  |  |  |
| **Study findings, limitations, generalisability, and current knowledge** | 26 | Report key findings, limitations, ethical or equity considerations not captured, and how these could affect patients, policy, or practice. | Page 10-14 |
| **Other relevant information** |  |  |  |
| **Source of funding** | 27 | Describe how the study was funded and any role of the funder in the identification, design, conduct, and reporting of the analysis | Page 2 |
| **Conflicts of interest** | 28 | Report authors conflicts of interest according to journal or International Committee of Medical Journal Editors requirements. | Page 2 |

The following table summarizes the main aspects of the simulation model and the cost-effectiveness analysis.

### Table S2. Summary of key design criteria for the model simulation and cost-effectiveness analysis.

| *Decision problem* | Cost-effectiveness of strategies to detect EC in women with postmenopausal bleeding using molecular testing of minimally invasive samples. |
| --- | --- |
| *Interventions* | Strategies using molecular testing of minimally invasive samples in early stages of postmenopausal bleeding diagnosis compared to the current diagnosis strategy. |
| *Model type* | Markov model cohort simulation implemented in R. |
| *Population* | Women with postmenopausal bleeding attending to gynaecological visits. |
| *Time horizon* | 35 years (from 50-year-old until 85-year-old or dead). |
| *Perspective* | NHS perspective including direct costs associated with screening, diagnosis, and treatment. |
| *Costs* | Euros in 2013 prices, but currently in force. |
| *Health benefits* | Effectiveness in QALYs. |
| *Discount rate* | 3% per year for costs and health benefits. Range 0-5% in sensitivity analysis |
| *Outputs* | Number of hysterectomies, lifetime expectancy, QALYs, endometrial cancer prevalence and incidence, mortality from endometrial cancer and the lifetime cost of screening, diagnosis, and treatment. |
| *Method of evaluation* | Cost-effectiveness analysis (€ per QALY gained) and deterministic and probabilistic sensitivity analyses for all parameters. |
| *Measure of CEA* | ICER defined as the difference in cost (€) between the two strategies divided by the difference in health (QALYs). The ICER corresponds to the incremental cost associated with one additional QALY gained. |
| *Threshold for cost-effectiveness* | ICER below 22,000-25,000 € per QALY. |

*EC, Endometrial cancer; CEA, Cost-effectiveness of analysis; ICER, Incremental cost-effectiveness ratio; NHS, National Health System; QALYs, Quality-adjusted life-years.*

## **Model**

As explained in the manuscript, we developed a homogeneous Markov cohort model with 6 mutually exclusive health states with diagnostic strategies diagrams for EC working as a decision tree: postmenopausal bleeding, detected endometrial cancer, no detected endometrial cancer, endometrial cancer survivor, death from endometrial cancer, and death from other causes. This closed model follows a single cohort of 50-year-old women (considered as the average age of menopause) using 1-year increments until they reach the age of 85 years (time horizon of 35 years) or die. All women start the model simulations with PMB and can move from one health state to another according to some transition probabilities. Women may die from EC in the cancer stage or from other causes in every health state and every cycle. The following figure shows the Markov model diagram that summarizes the states included in the study and transition probabilities.

### Figure S1. State transition diagram of the Markov Model.

**
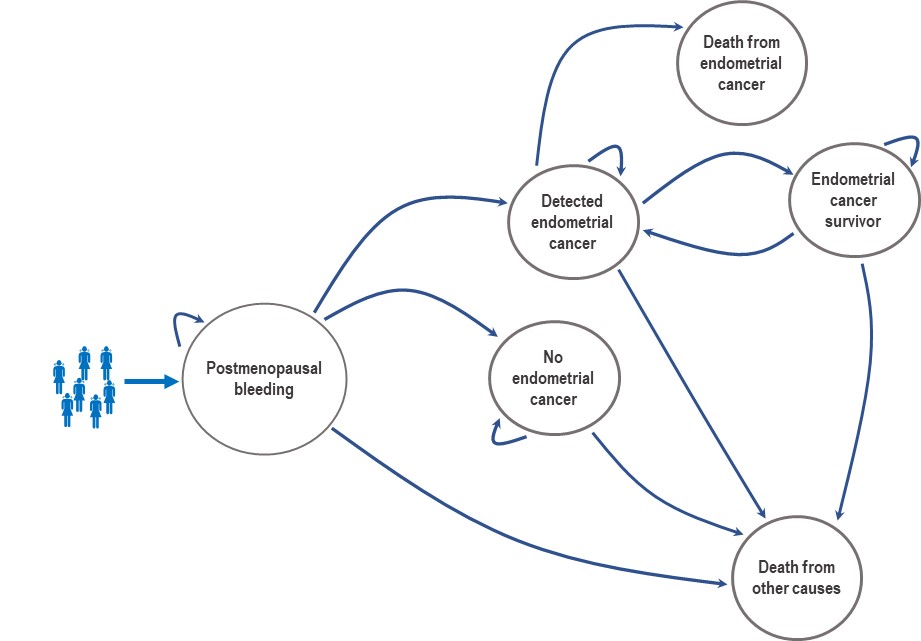
**

Some features of the model are detailed below:

1. The current strategy diagram includes a non-final node for a maximum re-evaluation at 12 months (Node: Re-evaluation in max 12m), as can be seen in the algorithm (Figure 2a of the manuscript). For modeling purposes, this node is represented in an auxiliary state not represented in the state transition diagram model (Figure 1 of the manuscript) because it is not a state per se. Since this node is only present in the current strategy diagram, this auxiliary state has no role in simulating the molecular strategy. As a result, women reaching this node need two cycles (a cycle is equivalent to one year) to reach the end of the diagram path. That is, women receive a TVU at the first cycle and those with an endometrial thickness below the 4mm threshold must wait until the next cycle for the re-evaluation. In the second cycle, women are re-evaluated and start at the next node (Node: Recurrent bleeding) and end at one of the final nodes in that same cycle. However, if the TVU measures an endometrial thickness above the 4mm threshold, the strategy continues until a final node that same cycle. As the molecular strategy diagram does not have this peculiarity, the diagram path always ends the same cycle it starts. In both diagrams, women who reach a final node of re-evaluation at 12 months (Node: Re-evaluation in 12m) return to the beginning of the diagram again until they complete the time horizon or die.
2. Two additional auxiliary states are also needed to track women with undetected EC. The first state is analogous to the PMB state, i.e., PMB women enter diagnostic strategies diagrams as usual, but with an undiagnosed EC because of a false negative result in previous cycles. Similarly, the second state is analogous to the above-mentioned auxiliary state (see paragraph I), but for those women whose EC had not previously detected. Women in these two states are indistinguishable from their analogous states except that their EC prevalence is equal to 1 and, when they are finally diagnosed, the EC will be at a more advanced stage.

# Input values, parameters, and assumptions

In this section of the appendix, we complement the information given in the Methods section and Table 1 of the manuscript and clarify some specific aspects.

## **Health input data**

As we used a homogeneous Markov model, transition probabilities and other model parameters remain fixed over the simulated time horizon, although some of them are age-dependent.

Health input data is based on the traditional histopathological classification of EC as well as FIGO classification due to the availability of data in published literature. Nevertheless, it has to be stated that in the previous years there have been many advances to improve the classification of EC and recently a novel molecular classification of EC has been proposed. Currently, both classifications co-exist. The novel molecular classification has the potential to improve patient management with the implementation of precision medicine as well as adapting the aggressiveness of the surgery and adjuvant treatment to avoid relapses. Nevertheless, both classifications of EC do not differ concerning the initial diagnostic procedures or perioperative management.

One-year global and age-specific prevalence rates of EC in general women aged ≥50 were extracted from Globocan 2020, and the global prevalence rate of EC in women with PMB was extracted from Clarke et al. 2018^2,3^. Annual probabilities of death from other causes by age group were calculated with the exponential transformation using mortality rates. Age-specific mortality rates from other causes in women with PMB were calculated using the age-specific female mortality rates from all causes and the age-specific female mortality rates from EC. Mortality rates from EC were extracted from Globocan 2020 and female mortality rates from all causes were extracted from Spanish Statistics National Institute^2,4^. Yearly probabilities of death for EC stages by age group were calculated with the exponential transformation using the survival rates^5^. We used 5-year survival rates from the USA 2018 for white women aged ≥45, as EC survival as data in Spain is not updated nor disaggregated by EC stage and age groups^6^.

1. Age-specific EC prevalence among women with PMB was estimated calculating the ratio between the global prevalence of EC in women with PMB and the global prevalence of EC in the general population of women aged ≥50. This ratio was applied to each age-specific prevalence group in the general population of women aged ≥50 to estimate the corresponding age-specific prevalence in women with PMB.
2. A small proportion of endometrial cancer cases are not detected at the end of the diagram path, and we assume that they will progress to the following stage in 1 year, where they are likely to be detected. Based on the expert consensus, the proportion of undetected EC cases in stage I that will progress in 1 year to stage II is set at 30%, 40% of undetected EC cases in stage II will progress to stage III, and 55% of EC cases in stage III will progress to stage IV.
3. We assume that women with endometrial cancer are cured after treatment but may have recurrence in the future according to the recurrence rates described in Table 1 of the manuscript.
4. The hazard ratio of women with BMI over 30 was roughly derived from the Bhaskaran et al. graph ^7^.
5. Since EC survival is not available for Spain, we also used 5-year survival rates from the SEER database 2018 for white women aged 45 and over^8^. We assumed that survival rates for stage II and III were the same and corresponding to the regional cancer. (See Table S3).
6. The distribution of cancer stage at diagnosis was extracted from the SEER database classified in localized, regional, distant stage and unknown. We assumed that localized cancer corresponds to stage I (tumour at primary site), regional cancer to stage II and III (assuming the tumour has grown outside the organ or has spread to reginal lymph nodes), and distant cancer to stage IV (when the tumour has spread distantly in the body)^8^. To separate the distribution of stage II and III, we used the article by Huijgens et al. where 10 cases were diagnosed in stage II and 24 in stage III^9^. Therefore, 29.4% of the total cases diagnosed in stage II and III were in stage II and 70.6% in stage III. We also assume that unstaged cases behave like as staged cases and are redistributed among the 3 stages in the same proportion that already existed so that the total adds up to 100% (see Table S2).

### Table S3. Proportion of cancer stage at diagnosis according to age groups.

| **Original data**  **from SEER** | Localized | Regional | | Distant | Unstaged | Total |
| --- | --- | --- | --- | --- | --- | --- |
| 55-64 | 0.72 | 0.191 | | 0.068 | 0.02 | 1.00 |
| 65-74 | 0.68 | 0.212 | | 0.085 | 0.023 | 1.00 |
| 75+ | 0.58 | 0.24 | | 0.109 | 0.074 | 1.00 |
| **Redistribution**  **of unstaged cases** | Localized | Regional | | Distant | Unstaged | Total |
| 55-64 | 0.735 | 0.195 | | 0.069 |  | 1.00 |
| 65-74 | 0.696 | 0.217 | | 0.087 |  | 1.00 |
| 75+ | 0.624 | 0.258 | | 0.117 |  | 1.00 |
| **Calculation**  **of stage II and III** | Stage  I | Stage II | Stage III | Stage  IV |  |  |
| 55-64 | 0.735 | 0.057 | 0.138 | 0.069 |  | 1.00 |
| 65-74 | 0.696 | 0.064 | 0.153 | 0.087 |  | 1.00 |
| 75+ | 0.624 | 0.076 | 0.182 | 0.117 |  | 1.00 |

1. Many input values of the model were age-dependent. However, overall values by stage were included in Table 1 of the manuscript as a summary. Table S3 shows the values for each age-group.

### Table S4. Summary of base case values according to age-group for model input parameters.

| **Parameters** | **Age-dependent input values** |
| --- | --- |
| Prevalence of EC in women aged 50+ (per 100,000) |  |
|  | 50-54y: 28.7  55-59y: 48.3  60-64y: 62.7  65-69y: 70  70y+: 71.2 |
| Proportion of EC cases (50+ years) |  |
| Stage I | 50-64y: 0.735  65-74y: 0.696  75y+: 0.624 |
| Stage II | 50-64y: 0.055  65-74y: 0.064  75y+: 0.076 |
| Stage III | 50-64y: 0.131  65-74y: 0.153  75y+: 0.182 |
| Stage IV | 50-64y: 0.079  65-74y: 0.087  75y+: 0.118 |
| Proportion of obese women aged 55-64 (BMI >30) | 50-54y: 0.152  55-64y: 0.213  65-74y: 0.269  75y+: 0.245 |
| 5-year survival rates and probability of death |  |
| EC stage I | 50-54y: 0.968  55-64y: 0.966  65-74y: 0.949  75y+: 0.934 |
| EC stage II | 50-54y: 0.818  55-64y: 0.752  65-74y: 0.690  75y+: 0.572 |
| EC stage III | 50-54y: 0.818  55-64y: 0.752  65-74y: 0.690  75y+: 0.572 |
| EC stage IV | 50-54y: 0.242  55-64y: 0.192  65-74y: 0.178  75y+: 0.097 |
| Probability of death from other causes |  |
|  | 50-54y: 0.001008  55-59y: 0.005437  60-64y: 0.005400  65-69y: 0.005350  70-74y: 0.005292  75y+: 0.062354 |

1. Table S4 shows age-specific female mortality rates from all causes^4^, age-specific female mortality from EC^2^ and annual death probabilities from all causes, EC and mortality from other causes calculated based on the exponential transformation of the mortality rates.

### Table S5. Mortality rates and probabilities according to age groups.

|  | **Rates** | | **Probabilities** | | |
| --- | --- | --- | --- | --- | --- |
| **Age-group** | **Global mortality** | **EC mortality** | **Global mortality** | **EC mortality** | **Mortality from other causes** |
| 25-29 | 0.000175850 | 0 | 0.000175835 | 0 | 0.0001758347 |
| 30-34 | 0.000236333 | 0 | 0.000236305 | 0 | 0.0002363053 |
| 35-39 | 0.000361056 | 0 | 0.000360990 | 0 | 0.0003609904 |
| 40-44 | 0.000587935 | 0.000002 | 0.000587763 | 0.000002000 | 0.0005857626 |
| 45-49 | 0.001055942 | 0.0000094 | 0.001055385 | 0.000009400 | 0.0010459849 |
| 50-54 | 0.001779161 | 0.000025 | 0.001777579 | 0.000025000 | 0.0017525791 |
| 55-59 | 0.002839309 | 0.000052 | 0.002835282 | 0.000051999 | 0.0027832833 |
| 60-64 | 0.004212524 | 0.000089 | 0.004203664 | 0.000088996 | 0.0041146675 |
| 65-69 | 0.005978143 | 0.000139 | 0.005960310 | 0.000138990 | 0.0058213195 |
| 70-74 | 0.009533141 | 0.000197 | 0.009487844 | 0.000196981 | 0.0092908639 |
| 75-79 | 0.017570219 | 0.000197 | 0.017416763 | 0.000196981 | 0.0172197826 |
| 80-84 | 0.037268715 | 0.000197 | 0.036582784 | 0.000196981 | 0.0363858037 |
| 85-89 | 0.080518584 | 0.000197 | 0.077362243 | 0.000196981 | 0.0771652626 |
| 90+ | 0.161296787 | 0.000197 | 0.148960544 | 0.000196981 | 0.1487635636 |

## **Utilities**

1. The utilities of cancer stages to calculate QALYs were extracted from Kwon et al. 2008 for stages I and II^10^. For stages III and IV, since they were not available in Kwon et al., they were extracted from Goldie et al. 2004^11^ which despite being in cervical cancer were consistent with the utilities of stages I and II.
2. The survival utility (women treated) for stage IV was also extracted from Goldie 2004 et al.^11^ (cervical cancer) because it was assumed that the treatment (hysterectomy) was similar in both cancers. For the other stages, we calculated the ratio between the survival utility in stage IV and the EC utility in stage IV and it was applied on the rest of EC utilities to obtain the corresponding survival utilities of stages I, II and III.

## **Cost input data**

Costs in euros were extracted from the latest revision of public prices corresponding to health services provided by the Catalan Institute of Health published in the Official Journal of the Government of Catalonia (DOGC)^12^. Although the prices were set up in 2013, they are currently in force, and for that reason, we assumed they are indexed in €2020. The costs for the different tests and interventions include other associated concepts such as outpatient gynecological visits and follow-up visits, phone consultations for the communication of results and other secondary interventions according to each strategy. Regarding the cost of the molecular test, a base value of 310€ was assumed according to other molecular tests available in the DOGC 2013 document^12^, although was widely varied in the sensitivity analysis.

# Calibration

Our model integrates biological, clinical, epidemiological, and economic data from various sources and from different periods that are mostly uncertain, and even non-existent in some cases. For example, the prevalence of women with endometrial cancer is estimated by different sources, such as Globocan. However, the prevalence of PMB women with endometrial cancer is unknown and can simply be estimated by making assumptions about the prevalence in the general population (see previous section). Performing a calibration with non-existent real data could introduce more uncertainty in the model.

For this reason, we have applied the principle of parsimony and presented the results in the main manuscript without calibrating the model since we cannot guarantee that the theoretical prevalence estimated by us is better than the prevalence estimated by the model with carefully selected input data. In these cases, as sensitivity analysis is more important than calibration, we have carried out a very extensive sensitivity analysis; deterministic and probabilistic, univariate and multivariate. In this analysis, we have changed all the parameters of the model, including those for calculating the prevalence (see Table S10).

We present here the calibrated model and the base case results. It should be noted that the results were practically the same with the calibrated model than without calibration. Only the prevalence of EC for women with PMB has been calibrated, since incidence, mortality and hysterectomies rates are not available in the scientific literature for the PMB women and would require greater assumptions than for the prevalence. We assume an age distribution for this prevalence equal to that of the general population but scaled on the value found in the literature (see Table1 of the main Manuscript and in the “Input values, parameters, and assumptions” section of the Supplementary Appendix). Parameters that have been modified in the calibration are the age-specific probabilities of developing cancer and the probabilities of recurrence for women cured from stage 1 endometrial cancer. These parameters have been modified by a range of +-25% of their initial value.

The method used for calibration is the limited memory variant of the Broyden–Fletcher–Goldfarb–Shanno (L-BFGS) algorithm from the quasi-Newton family optimization methods. These are algorithms for finding local optima of functions, which are based on Newton’s method of finding stationary points of functions^13^.

### Table S6. Prevalence of EC for women with PMB.

| Age-group | Theorical prevalence | Prevalence without  model calibration | Prevalence with  model calibration |
| --- | --- | --- | --- |
| 50-54 | 0.04822781 | 0.03458316 | 0.04312563 |
| 55-59 | 0.08116388 | 0.07057034 | 0.08104754 |
| 60-64 | 0.10536181 | 0.09123084 | 0.10583851 |
| 65-69 | 0.11762881 | 0.09869087 | 0.11568249 |
| 70-74 | 0.11964530 | 0.09945004 | 0.11588222 |
| 75-79 | 0.11964530 | 0.09853164 | 0.11459087 |
| 80-84 | 0.11964530 | 0.09764194 | 0.11385536 |

### Figure S2. Theorical prevalence data and uncalibrated model-predicted values.

**
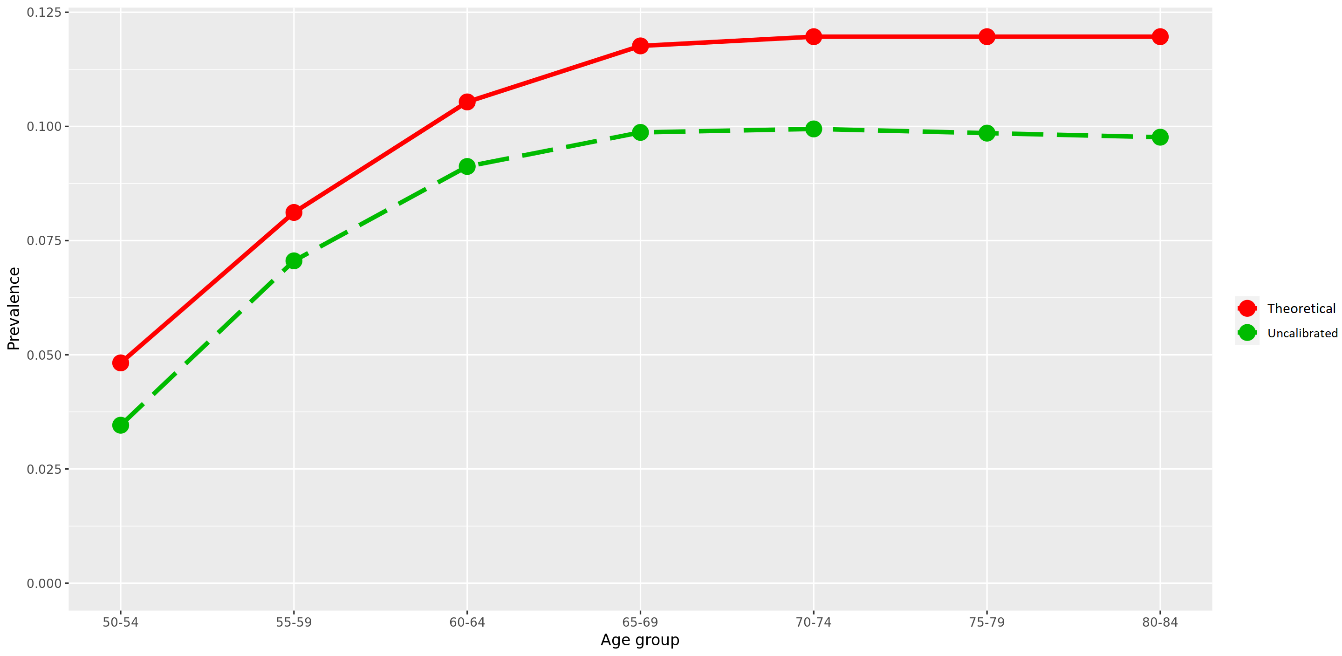
**

### Figure S3. Theorical prevalence data and calibrated model-predicted values.

**
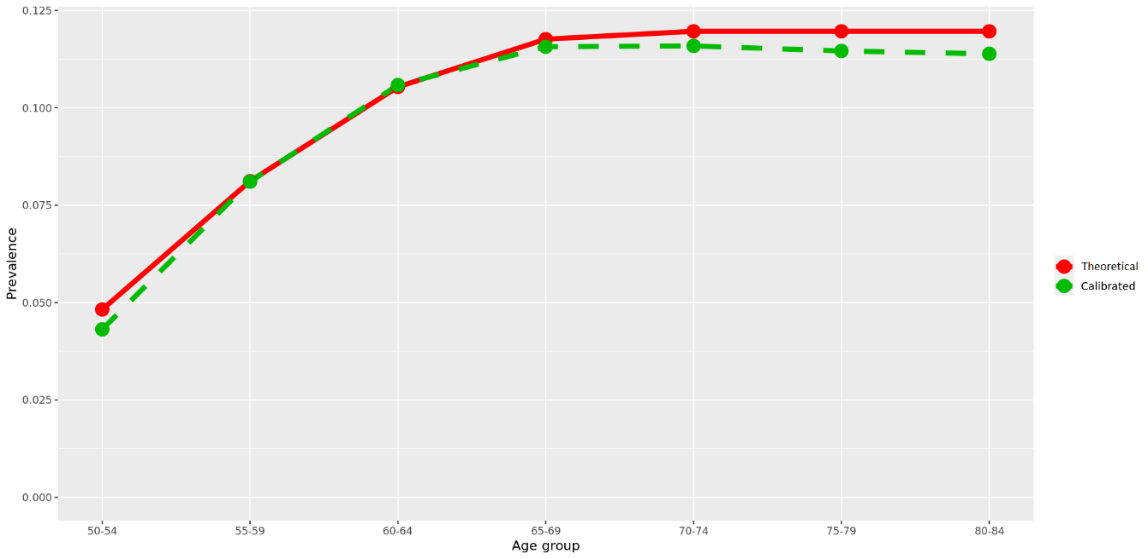
**

As seen in the graphs, the probability of having endometrial cancer in PMB women increases in the calibrated model, and therefore will favor the molecular strategy as will be seen in the following results.

Below we present the main outcomes and the baseline cost-effectiveness analysis with the calibrated and uncalibrated model (Table S7):

**Table S7. Cost-effectiveness analysis with the calibrated and uncalibrated model.**

| Cost-effectiveness analysis | Not calibrated model | Calibrated model |
| --- | --- | --- |
| Cost of the current strategy (€) | 12,124.14 | 11,929.44 |
| Cost of the molecular strategy (€) | 10,726.06 | 10,410.73 |
| ∆Cost (€) | 1,398.08 | 1,518.71 |
| Effectiveness of the current strategy (QALY) | 17.24976 | 17.03332 |
| Effectiveness of the molecular strategy (QALY) | 17.29218 | 17.08607 |
| ∆Effectiveness (QALY) | -0.04242 | -0.05275 |
| ICER (€/QALY) | -32,952 | -28,788 |

In the base case analysis, the molecular strategy increases the effectiveness compared to the current strategy and remains cost-saving. The sensitivity analysis also produces the same results.

# Sensitivity analysis

One-way DSA was divided into two groups of parameters. Those in which we know the possible range of values based on published data and expert opinion (Table 1), and those in which the range of values is unknown and were varied by ±5% of the base value. One-way, two-way and multivariate (varying several variables simultaneously) PSA were also performed for the most influential parameters in the results.

The parameters of the distributions were set with the mean as the base-case value and deviations at a tenth and a sixth of the base-case value. When the values of these deviations were not valid (e.g. beta distributions with a mean value close to one), the value was truncated to its maximum allowed value. Cost-effectiveness planes from PSA were used to visually represent incremental health outcomes and incremental costs between the molecular and the usual practice strategy. The cost-effectiveness acceptability curve was built to indicate if the optimal strategy changed over the increasing WTP from $0 to €50,000 per year of life gained.

As recommended by cost-effectiveness guidelines, the discount rate was varied in the sensitivity analysis to 0% and 5%^14^.

# Results

The graphs below show the annual number of hysterectomies (in cancers and non-cancers) and the number of cancer cases (detected and undetected) by age-group and overall, assuming an initial cohort of 100,000 women.

### Figure S4. Annual number of hysterectomies and cancers by age group (A-E).

| A. Age-group 50-54 years | B. Age-group 55-59 years. |
| --- | --- |
| 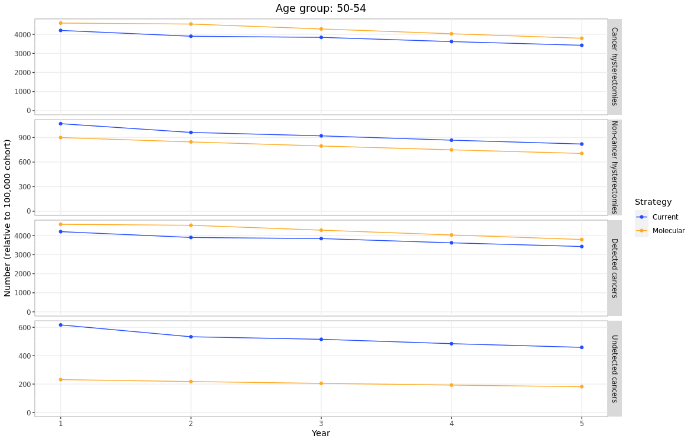 | 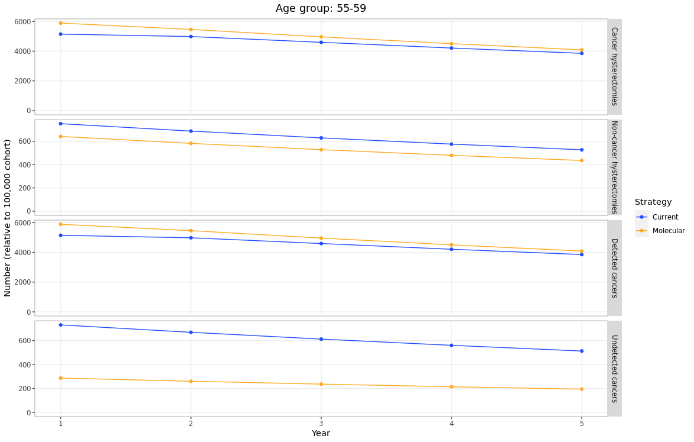 |
| C. Age-group 60-64 years. | D. Age-group 65-69 years. |
| 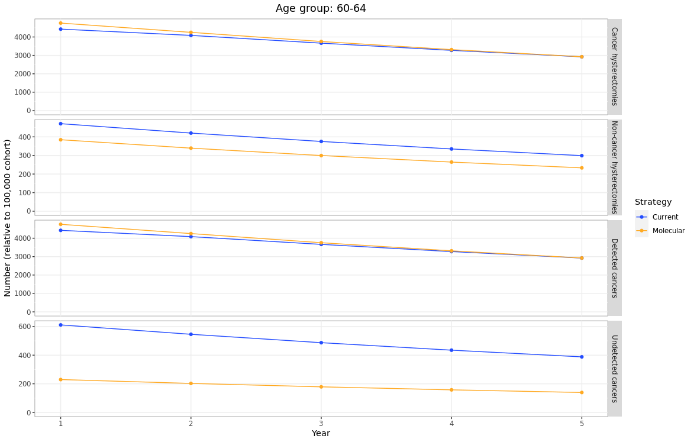 | 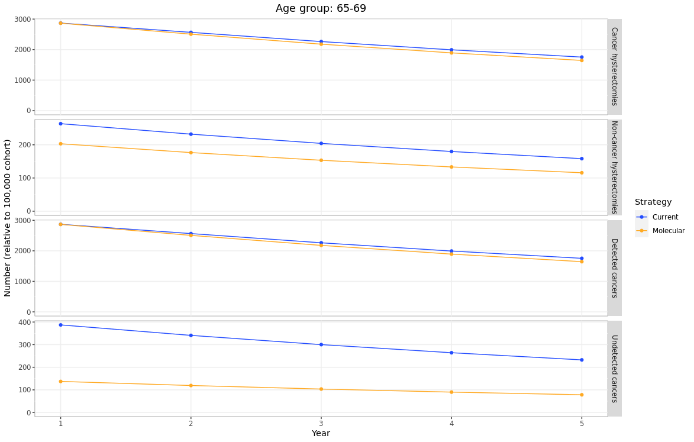 |
| E. Age-group 70+ years. |  |
| 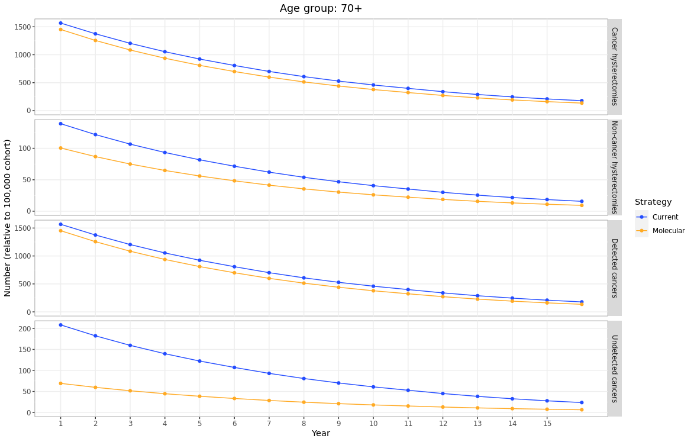 |  |

The following table includes annual averages of hysterectomies and cancers by strategy and age-group, assuming an initial cohort of 100,000 women. The row of overall cases also corresponds to annual averages, so the sum of the columns by age must not add up to the total.

### Table S8. Mean annual number of hysterectomies and cancers by age groups and overall.

| **Age group** | **Strategy** | **Hysterectomies** | | | **Cancers** | | | **Figure S4** |
| --- | --- | --- | --- | --- | --- | --- | --- | --- |
|  |  | **Total** | **With no cancer** | **With cancer** | **Total** | **Detected** | **Undetected** |  |
|  |  | **n** | **n** | **n** | **n** | **n** | **n** |  |
| *50-54* | Current | 4,761 | 964 | 3,797 | 4,354 | 3,797 | 557 | A |
|  | Molecular | 4,926 | 807 | 4,119 | 4,326 | 4,119 | 207 |  |
| *55-59* | Current | 5,274 | 671 | 4,604 | 5,279 | 4,604 | 675 | B |
|  | Molecular | 5,395 | 545 | 4,850 | 5,095 | 4,850 | 244 |  |
| *60-64* | Current | 4,128 | 407 | 3,722 | 4,267 | 3,722 | 546 | C |
|  | Molecular | 4,055 | 315 | 3,740 | 3,928 | 3,740 | 188 |  |
| *65-69* | Current | 2,543 | 224 | 2,320 | 2,660 | 2,320 | 340 | D |
|  | Molecular | 2,359 | 164 | 2,196 | 2,306 | 2,196 | 110 |  |
| *70+* | Current | 2,382 | 206 | 2,176 | 2,495 | 2,176 | 319 | E |
|  | Molecular | 1,997 | 136 | 1,861 | 1,954 | 1,861 | 94 |  |
| **Overall** | **Current** | **19,090** | **2,472** | **16,618** | **19,056** | **16,618** | **2,437** |  |
|  | **Molecular** | **18,732** | **1,966** | **16,766** | **17,610** | **16,766** | **844** |  |

## **Sensitivity Analysis**

One-way DSA for the parameters in which the range of values was uncertain were varied by ±5% of the base value and represented in a tornado diagram. The manuscript only presents the graph by the most variable parameters given the extent of the graph and the little variability of some of the parameters. Figure S1 shows the full tornado diagram in the results section of the appendix.

### Table S9. One-way deterministic sensitivity analysis of parameters with known ranges. Cost-effectiveness in € per QALY gained.

| **Parameter** | **Base value** | **Rank** | **Cost (€)** | **∆Cost (€)** | **Effectiveness (QALY)** | **∆Effectiveness (QALY)** | **ICER (€/QALY)** |
| --- | --- | --- | --- | --- | --- | --- | --- |
| **Molecular strategy (base)** |  |  | **10,721** | **-1,392** | **17.2854** | **0.0433** | **CS** |
| Cost of hysterectomy, EC stage I (€) | 5,367 | 3,817 | 9,892 | -1,239 | 17.2922 | 0.0424 | CS |
|  |  | 6,917 | 11,559 | -1,556 | 17.2922 | 0.0424 | CS |
| Cost of hysterectomy, EC stage II-IV (€) | 10,606 | 7,543 | 10,242 | -1,306 | 17.2922 | 0.0424 | CS |
|  |  | 13,669 | 11,209 | -1,490 | 17.2922 | 0.0424 | CS |
| Cost of treatment (€) | 4,326 | 3,473 | 10,579 | -1,308 | 17.2922 | 0.0424 | CS |
|  |  | 8,653 | 11,467 | -1,851 | 17.2922 | 0.0424 | CS |
| HR BMI | 3.00 | 2.50 | 10,725 | -1,398 | 17.2922 | 0.0424 | CS |
|  |  | 4.00 | 10,726 | -1,397 | 17.2922 | 0.0425 | CS |
| Prevalence of PMB in women with EC | 0.91 | 0.87 | 10,725 | -1,365 | 17.2919 | 0.0478 | CS |
|  |  | 0.93 | 10,726 | -1,414 | 17.2923 | 0.0398 | CS |
| Probability of EC in PMB | 0.09 | 0.08 | 10,887 | -1,331 | 17.3899 | 0.0370 | CS |
|  |  | 0.11 | 10,443 | -1,506 | 17.1300 | 0.0534 | CS |
| Probability of pipelle success (insertion) | 0.92 | 0.89 | 10,747 | -1,376 | 17.2801 | 0.0310 | CS |
|  |  | 0.94 | 10,711 | -1,412 | 17.3003 | 0.0501 | CS |
| Probability of pipelle success (tissue) | 0.87 | 0.86 | 10,732 | -1,391 | 17.2912 | 0.0417 | CS |
|  |  | 0.90 | 10,705 | -1,418 | 17.2950 | 0.0446 | CS |

| Sensitivity of molecular test (Pap-smear) | 0.78 | 0.75 | 10,719 | -1,404 | 17.2884 | 0.0387 | CS |
| --- | --- | --- | --- | --- | --- | --- | --- |
|  |  | 0.85 | 10,741 | -1,383 | 17.3009 | 0.0512 | CS |
| Sensitivity of molecular test (pipelle) | 0.96 | 0.92 | 10,722 | -1,401 | 17.2901 | 0.0403 | CS |
|  |  | 0.98 | 10,727 | -1,396 | 17.2932 | 0.0435 | CS |
| Sensitivity of pipelle (PMB) | 0.94 | 0.84 | 10,713 | -1,384 | 17.2751 | 0.0447 | CS |
|  |  | 0.99 | 10,732 | -1,404 | 17.3007 | 0.0413 | CS |
| Specificity of molecular test (Pap-smear) | 0.97 | 0.83 | 10,597 | -1,526 | 17.1348 | -0.1150 | 13,271 |
|  |  | 1.00 | 10,754 | -1,369 | 17.3280 | 0.0782 | CS |
| Specificity of molecular test (pipelle) | 0.94 | 0.79 | 10,689 | -1,434 | 17.2785 | 0.0288 | CS |
|  |  | 0.99 | 10,738 | -1,385 | 17.2968 | 0.0470 | CS |
| Specificity of pipelle | 0.99 | 0.98 | 10,561 | -1,508 | 17.1823 | 0.0397 | CS |
|  |  | 1.00 | 10,901 | -1,277 | 17.4098 | 0.0454 | CS |
| Discount | 0.03 | 0.00 | 13,655 | -1,975 | 26.6561 | 0.0978 | CS |
|  |  | 0.05 | 9,296 | -1,146 | 13.5236 | 0.0240 | CS |

*BMI, body-mass index; CS, cost-saving; DSA, Deterministic Sensitivity Analysis; EC, endometrial cancer; ICER, incremental cost effectiveness ratio; PMB, postmenopausal bleeding; QALY, quality-adjusted life-years.*

∆Cost: Incremental cost = cost of molecular strategy - cost of current strategy. ∆Effectiveness: Incremental effectiveness = effectiveness of molecular strategy - effectiveness of current strategy. ICER = Incremental cost / Incremental Effectiveness.

### Table S10. One-way deterministic sensitivity analysis of parameters with known ranges. Cost-effectiveness in € per LY gained.

| **Parameter** | **Base value** | **Rank** | **Cost (€)** | **∆Cost (€)** | **Effectiveness (LY)** | **∆Effectiveness (LY)** | **ICER (€/LY)** |
| --- | --- | --- | --- | --- | --- | --- | --- |
| Molecular strategy (base) |  |  | 10,721 | -1,392 | 19.9106 | 0.0367 | CS |
| Cost of hysterectomy, EC stage I (€) | 5,367 | 3,817 | 9,892 | -1,239 | 19.9163 | 0.0359 | CS |
|  |  | 6,917 | 11,559 | -1,556 | 19.9163 | 0.0359 | CS |
| Cost of hysterectomy, EC stage II-IV (€) | 10,606 | 7,543 | 10,242 | -1,306 | 19.9163 | 0.0359 | CS |
|  |  | 13,669 | 11,209 | -1,490 | 19.9163 | 0.0359 | CS |
| Cost of treatment (€) | 4,326 | 3,473 | 10,579 | -1,308 | 19.9163 | 0.0359 | CS |
|  |  | 8,653 | 11,467 | -1,851 | 19.9163 | 0.0359 | CS |
| HR BMI | 3.00 | 2.50 | 10,725 | -1,398 | 19.9163 | 0.0358 | CS |
|  |  | 4.00 | 10,726 | -1,397 | 19.9163 | 0.0359 | CS |
| Prevalence of PMB in women with EC | 0.91 | 0.87 | 10,725 | -1,365 | 19.9161 | 0.0405 | CS |
|  |  | 0.93 | 10,726 | -1,414 | 19.9164 | 0.0336 | CS |
| Probability of EC in PMB | 0.09 | 0.08 | 10,887 | -1,331 | 19.9642 | 0.0304 | CS |
|  |  | 0.11 | 10,443 | -1,506 | 19.8360 | 0.0460 | CS |
| Probability of pipelle success (insertion) | 0.92 | 0.89 | 10,747 | -1,376 | 19.9197 | 0.0434 | CS |
|  |  | 0.94 | 10,711 | -1,412 | 19.9140 | 0.0308 | CS |
| Probability of pipelle success (tissue) | 0.87 | 0.86 | 10,732 | -1,391 | 19.9150 | 0.0360 | CS |
|  |  | 0.90 | 10,705 | -1,418 | 19.9203 | 0.0356 | CS |
| Sensitivity of molecular test (Pap-smear) | 0.78 | 0.75 | 10,719 | -1,404 | 19.9132 | 0.0327 | CS |
|  |  | 0.85 | 10,741 | -1,383 | 19.9236 | 0.0431 | CS |

| Sensitivity of molecular test (pipelle) | 0.96 | 0.92 | 10,722 | -1,401 | 19.9145 | 0.0341 | CS |
| --- | --- | --- | --- | --- | --- | --- | --- |
|  |  | 0.98 | 10,727 | -1,396 | 19.9172 | 0.0368 | CS |
| Sensitivity of pipelle (PMB) | 0.94 | 0.84 | 10,713 | -1,384 | 19.9021 | 0.0380 | CS |
|  |  | 0.99 | 10,732 | -1,404 | 19.9234 | 0.0348 | CS |
| Specificity of molecular test (Pap-smear) | 0.97 | 0.83 | 10,597 | -1,526 | 19.9673 | 0.0868 | CS |
|  |  | 1.00 | 10,754 | -1,369 | 19.9048 | 0.0243 | CS |
| Specificity of molecular test (pipelle) | 0.94 | 0.79 | 10,689 | -1,434 | 19.9207 | 0.0403 | CS |
|  |  | 0.99 | 10,738 | -1,385 | 19.9148 | 0.0344 | CS |
| Specificity of pipelle | 0.99 | 0.98 | 10,561 | -1,508 | 19.9519 | 0.0346 | CS |
|  |  | 1.00 | 10,901 | -1,277 | 19.8784 | 0.0372 | CS |
| Discount | 0.03 | 0.00 | 13,655 | -1,975 | 31.1010 | 0.0751 | CS |
|  |  | 0.05 | 9,296 | -1,146 | 15.4420 | 0.0223 | CS |

*BMI, body-mass index; CS, cost-saving; DSA, Deterministic Sensitivity Analysis; EC, endometrial cancer; ICER, incremental cost effectiveness ratio; PMB, postmenopausal bleeding; LY, life-years.*

∆Cost: Incremental cost = cost of molecular strategy - cost of current strategy. ∆Effectiveness: Incremental effectiveness = effectiveness of molecular strategy - effectiveness of current strategy. ICER = Incremental cost / Incremental Effectiveness.

### Figure S5. One-way deterministic sensitivity analysis for selected parameters with unknown ranges. Parameter values were varied by ±5% of the base value.


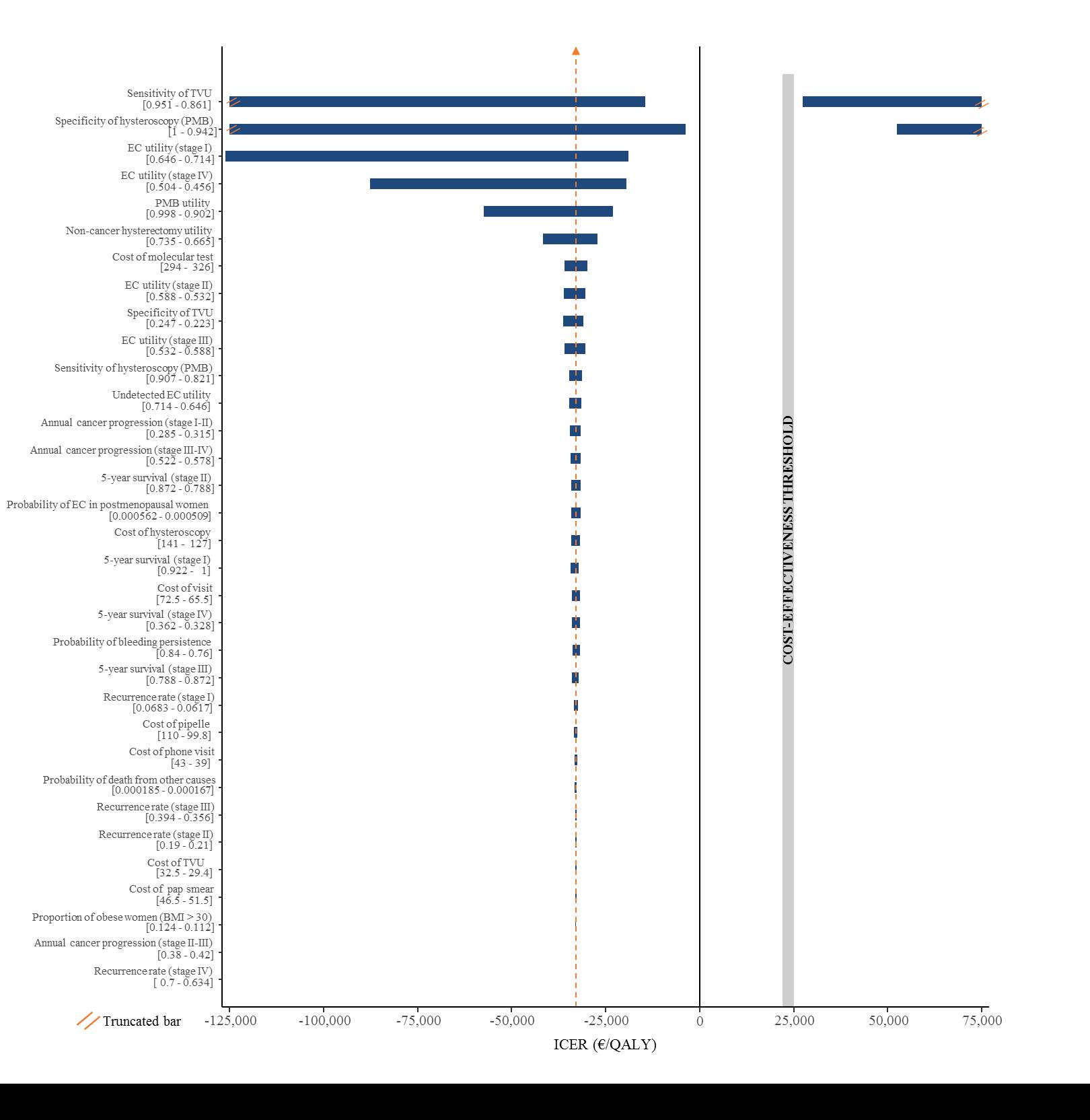


The following graphs depicts the cost-effectiveness acceptability curves showing the probability for the molecular strategy to be cost-effective at different willingness to pay values and costs of the molecular test (Figure S6) and the incremental cost-effectiveness plane for the base-case and deterministic sensitivity analysis for parameters with known ranges (Figure S7).

### Figure S6. Cost-effectiveness acceptability at different willingness to pay values and costs of the molecular test.

*QALY, quality-adjusted life-years; WTP, willingness to pay.*

Probability of cost-effectiveness at different WTP thresholds. Each line represents the percentage of cost-effective simulations for each molecular test cost. The values in brackets represent the range of cost-effectiveness simulations when WTP is set at 22.000-25.000 €/QALY. As the molecular test cost increases, the number of cost-effective simulations decrease and the WTP threshold needs to increase to remain cost-effective.

### Figure S7. Incremental cost-effectiveness plane for parameters with known ranges.


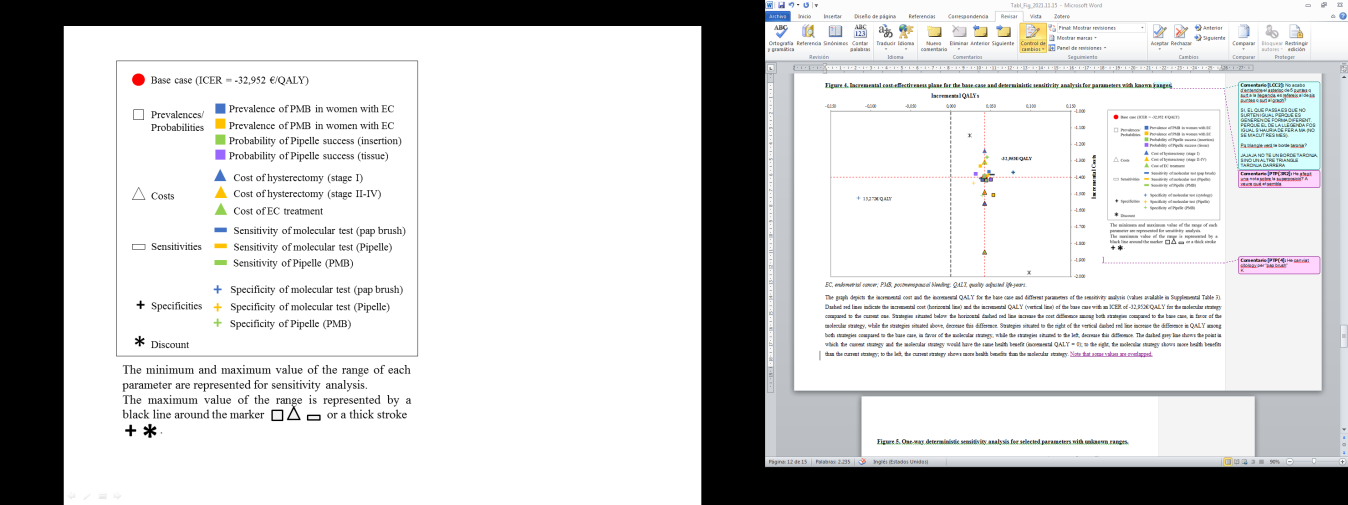

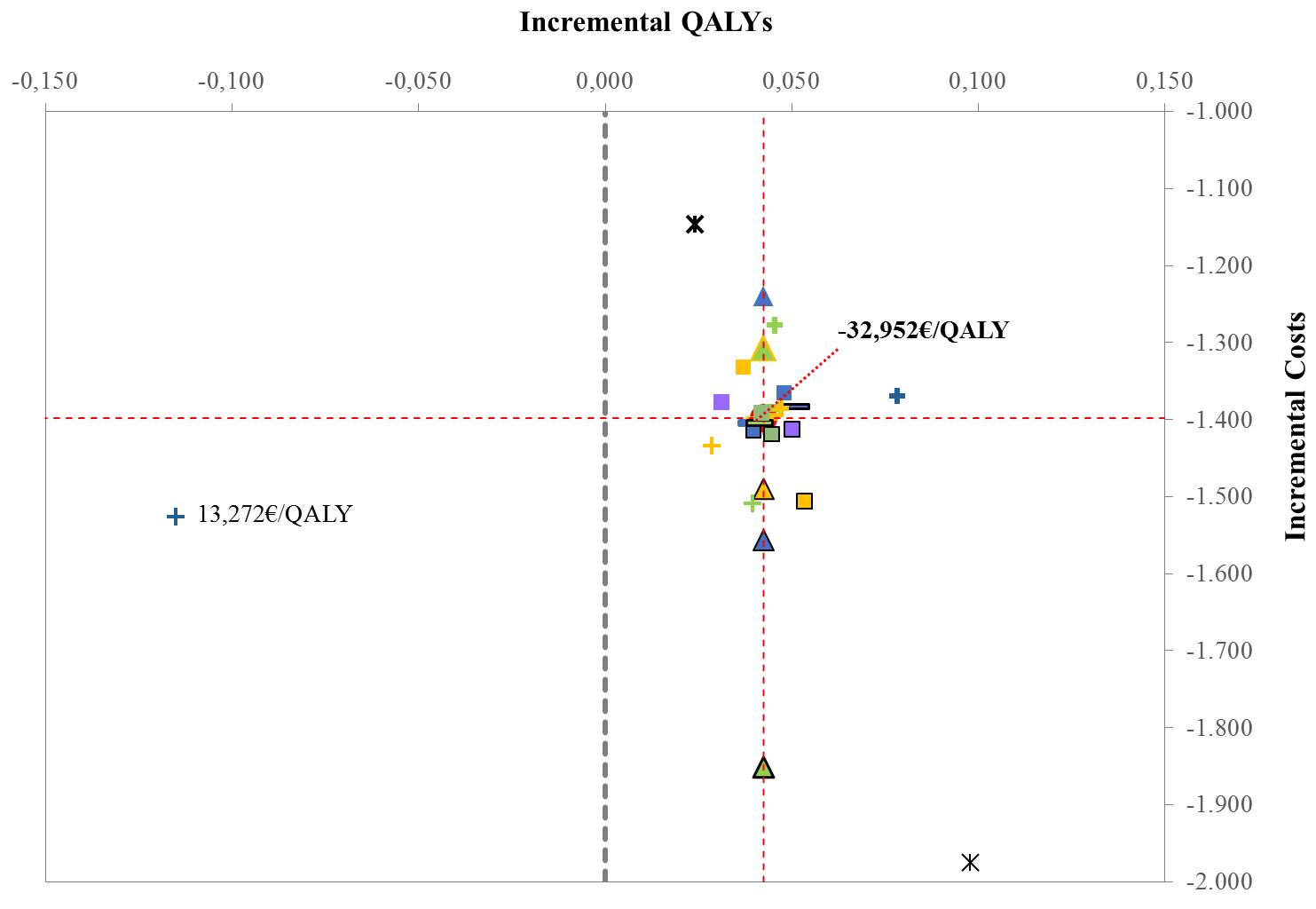


*EC, endometrial cancer; PMB, postmenopausal bleeding; QALY, quality adjusted life-years.*

The graph depicts the incremental cost and the incremental QALY for the base case and different parameters of the sensitivity analysis (values available in Supplemental Table 3). Dashed red lines indicate the incremental cost (horizontal line) and the incremental QALY (vertical line) of the base case with an ICER of -32,952€/QALY for the molecular strategy compared to the current one. Strategies situated below the horizontal dashed red line increase the cost difference among both strategies compared to the base case, in favor of the molecular strategy, while the strategies situated above, decrease this difference. Strategies situated to the right of the vertical dashed red line increase the difference in QALY among both strategies compared to the base case, in favor of the molecular strategy, while the strategies situated to the left, decrease this difference. The dashed grey line shows the point in which the current strategy and the molecular strategy would have the same health benefit (incremental QALY = 0); to the right, the molecular strategy shows more health benefits than the current strategy; to the left, the current strategy shows more health benefits than the molecular strategy.

### Figures S8- S57. Univariate probability sensitivity analysis (PSA) for all parameters using as standard deviation (SD) a tenth of the base case value (SD= base value/10).

| **Figure S8. PSA for the cost of Pap-smear.**  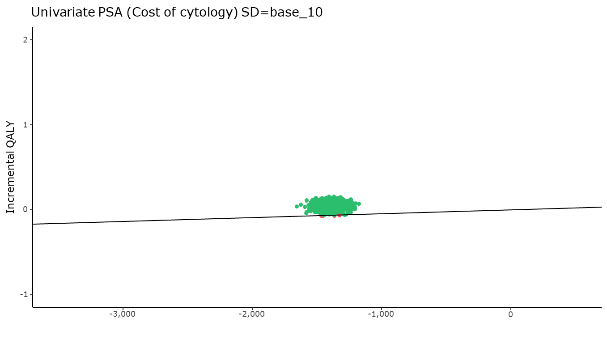 | **Figure S9. PSA for the cost of the initial visit.**  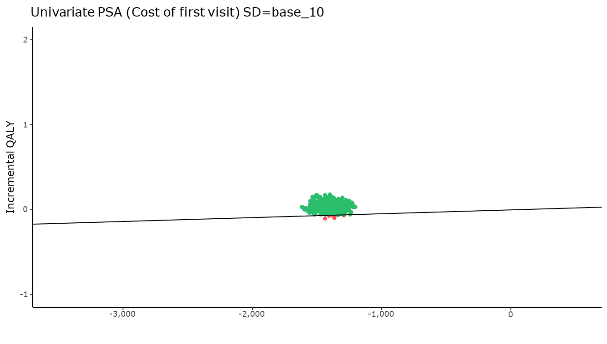 |
| --- | --- |
| **Figure S10. PSA for the cost of successive visits.**  **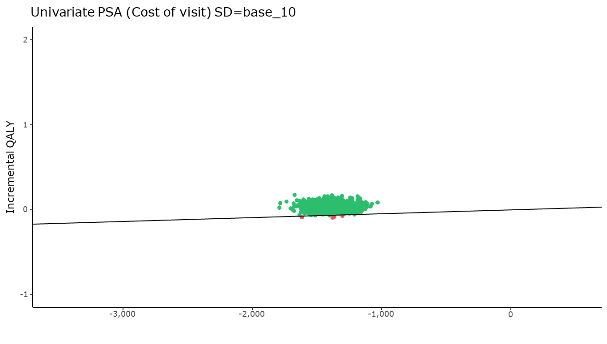** | **Figure S11. PSA for the cost of telephone visit.**  **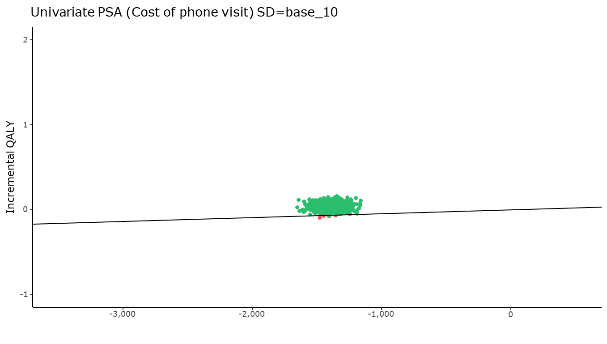** |
| **Figure S12. PSA for the cost of hysterectomy, EC stage I, in €.**  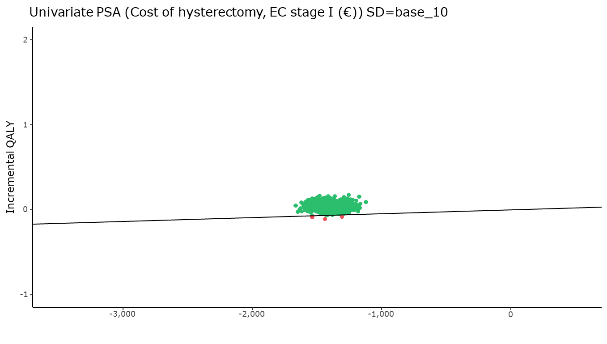 | **Figure S13. PSA for the cost of hysterectomy, EC stage II-IV, in €.**  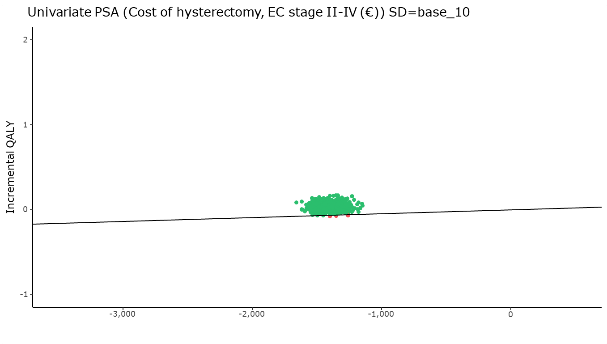 |
| **Figure S114. PSA for the cost of hysteroscopy.**  **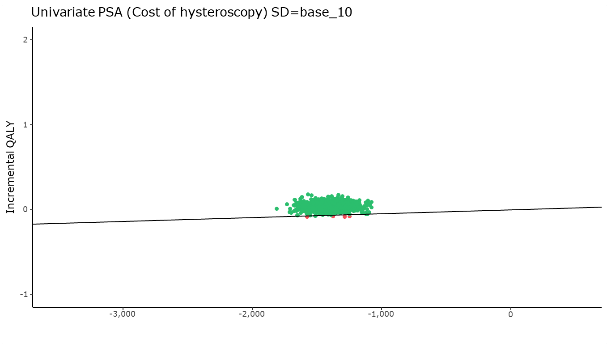** | **Figure S15. PSA for the cost of the molecular test.**  **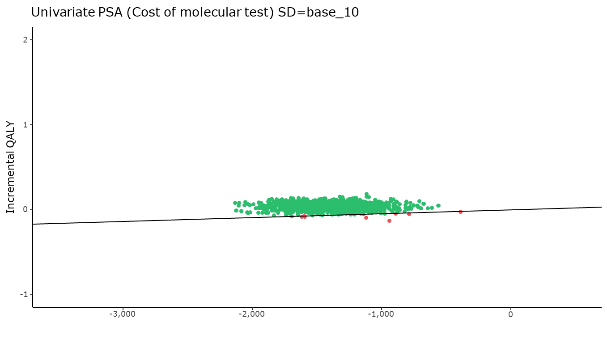** |

| **Figure S16. PSA for the cost of the pipelle.**  **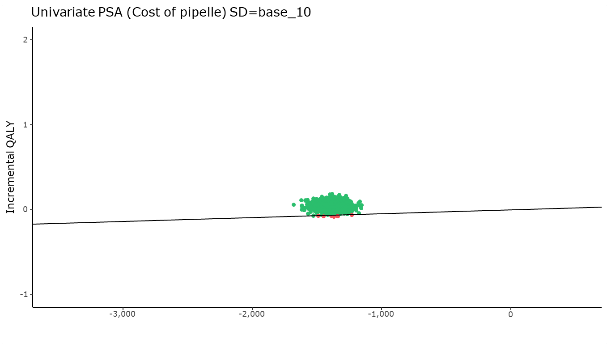** | **Figure S17. PSA for the cost of EC treatment, in €.**  **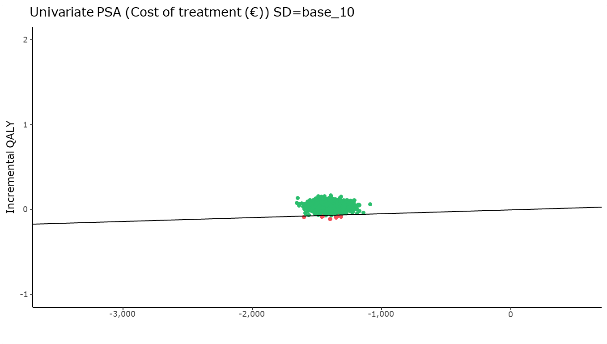** |
| --- | --- |
| **Figure S18. PSA for the cost of TVU.**  **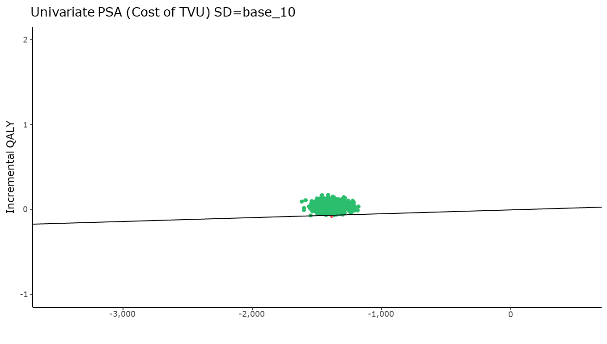** | **Figure S19. PSA for the proportion of obese women (BMI >30).**  **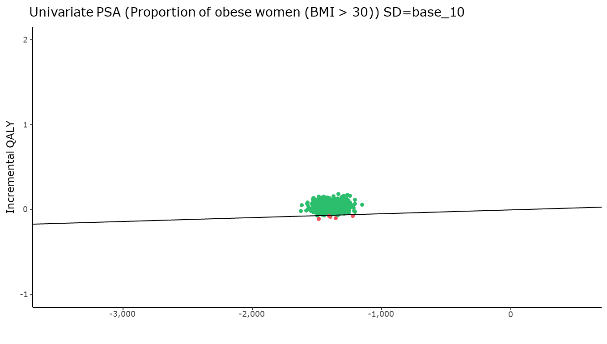** |
| **Figure S20. PSA for the hazard ratio of EC risk in obese women.**  **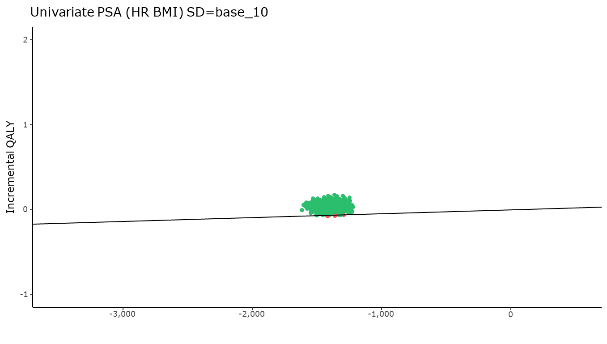** | **Figure S21. PSA for the probability of bleeding persistence.**  **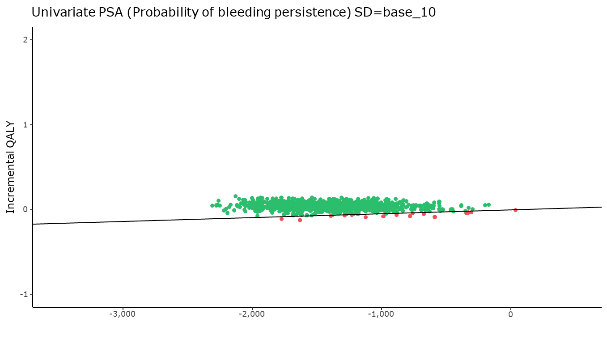** |
| **Figure S22. PSA for the prevalence of PMB in women with EC.**  **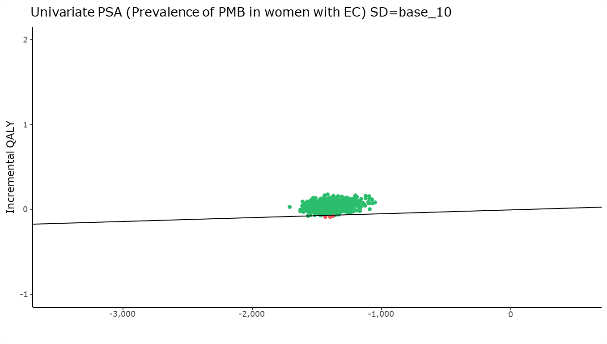** | **Figure S23. PSA for the probability of EC in women with PMB.**  **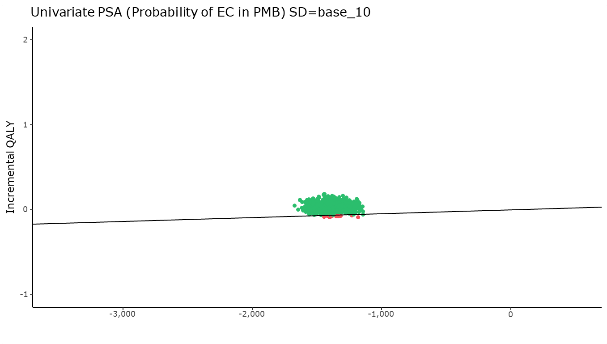** |

| **Figure S24. PSA for EC probability among premenopausal women.**  **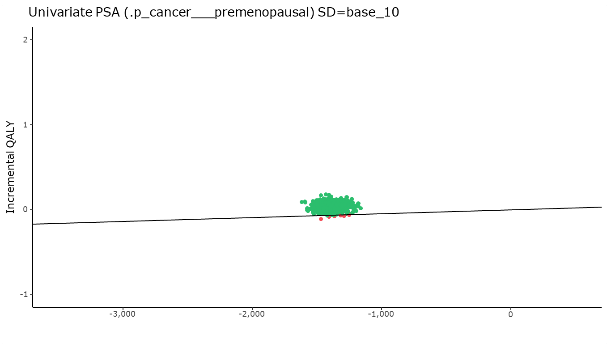** | **Figure S25. PSA for the probability of EC in postmenopausal women.**  **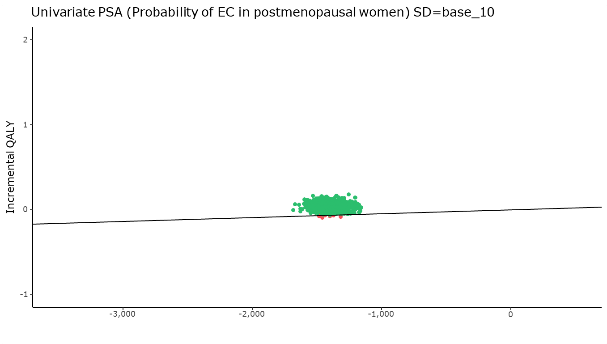** |
| --- | --- |
| **Figure S26. PSA for the probability of successful pipelle insertion.**  **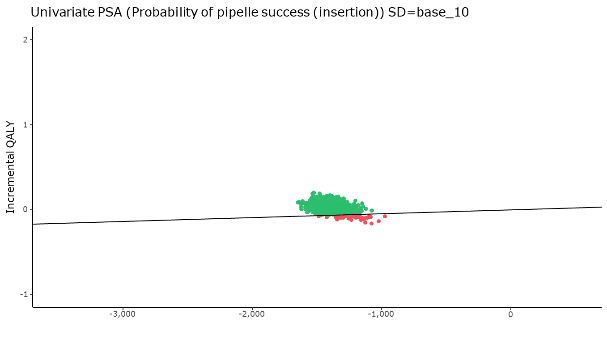** | **Figure S27. PSA for the probability of successful pipelle insertion in nulliparous women.**  **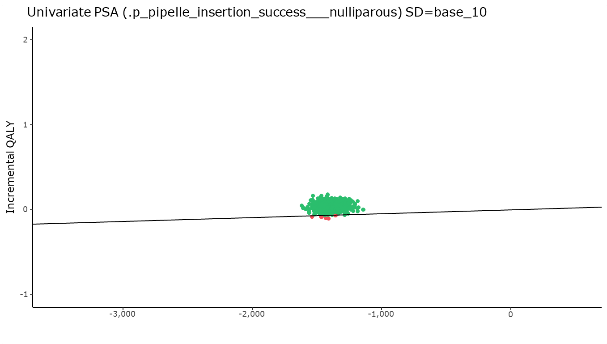** |
| **Figure S28. PSA for the probability of successful tissue obtention using pipelle.**  **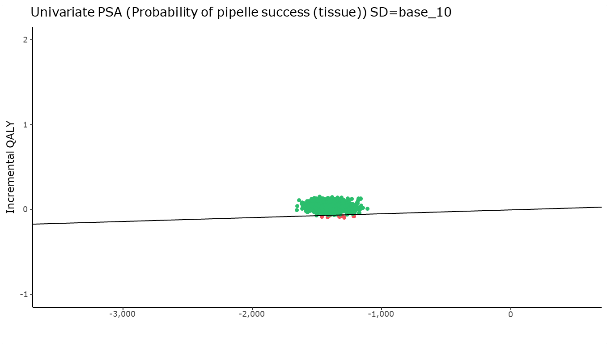** | **Figure S29. PSA for the annual probability of EC progression (stage I-II).**  **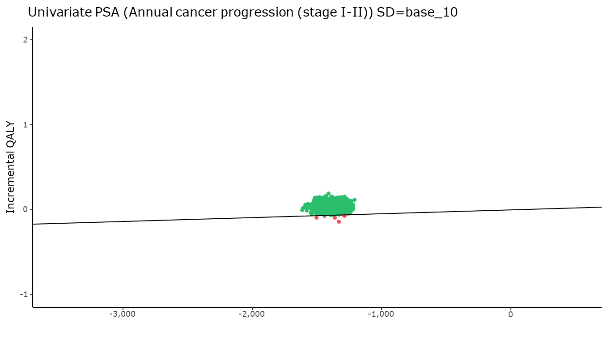** |
| **Figure S30. PSA for the annual probability of EC progression (stage II-III).**  **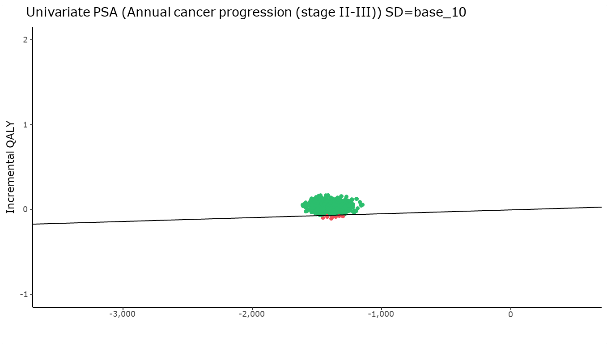** | **Figure S31. PSA for the annual probability of EC progression (stage III-IV).**  **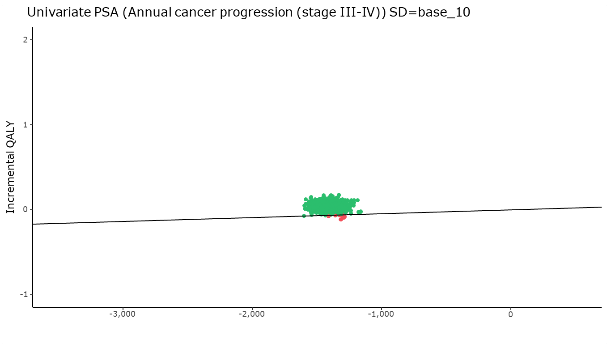** |

| **Figure S32. PSA for the recurrence rate (stage I).**  **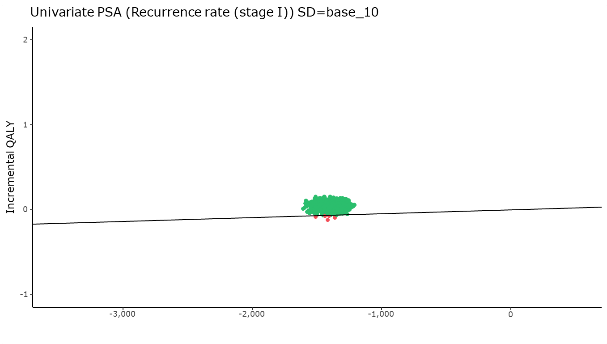** | **Figure S33. PSA for the recurrence rate (stage II).**  **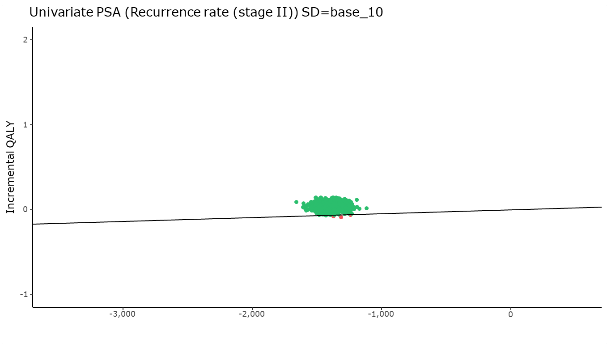** |
| --- | --- |
| **Figure S34. PSA for the recurrence rate (stage III).**  **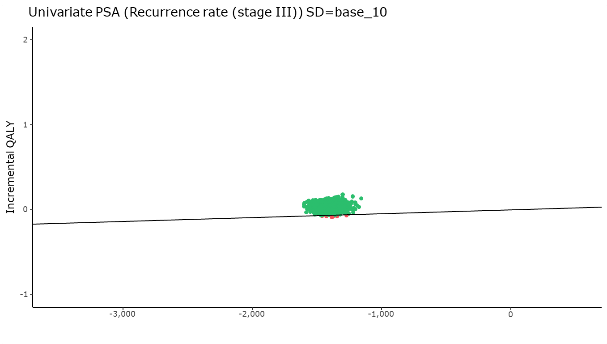** | **Figure S35. PSA for the recurrence rate (stage IV).**  **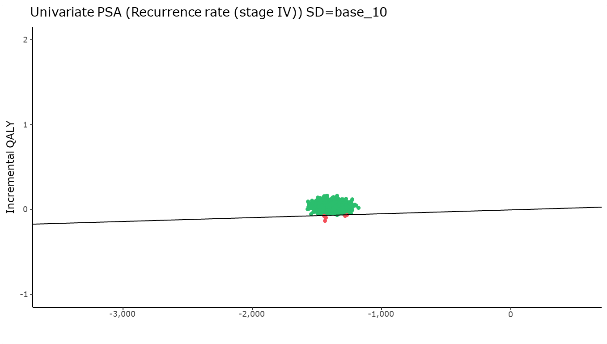** |
| **Figure S36. PSA for the sensitivity of hysteroscopy.**  **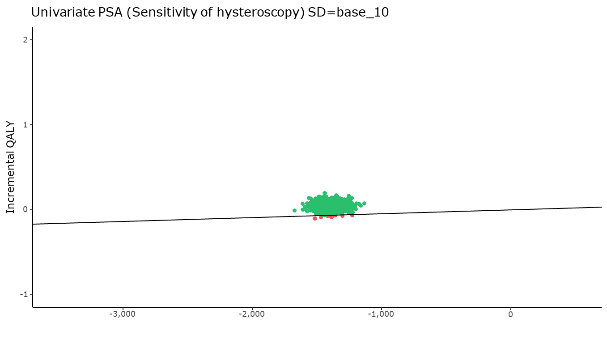** | **Figure S37. PSA for the specificity of hysteroscopy.**  **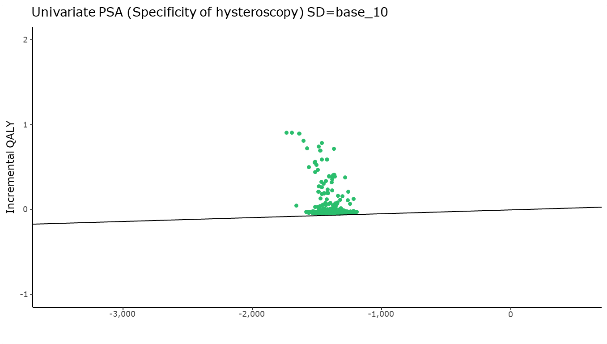** |
| **Figure S38. PSA for the sensitivity of the molecular test on pap brush.**  **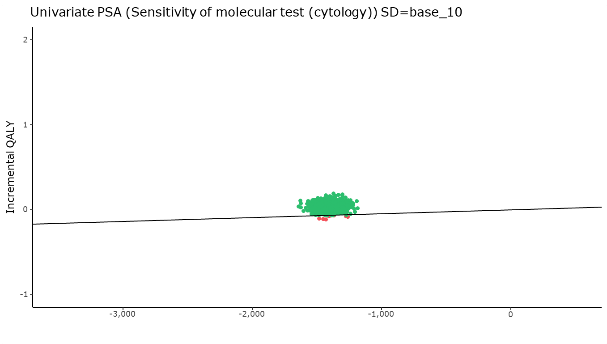** | **Figure S39. PSA for the specificity of the molecular test on pap brush.**  **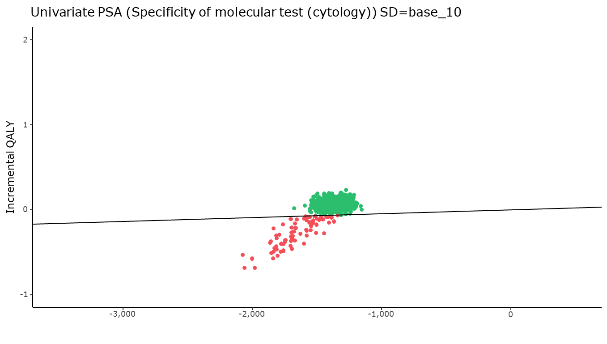** |

| **Figure S40. PSA for the sensitivity of the molecular test on pipelle.**  **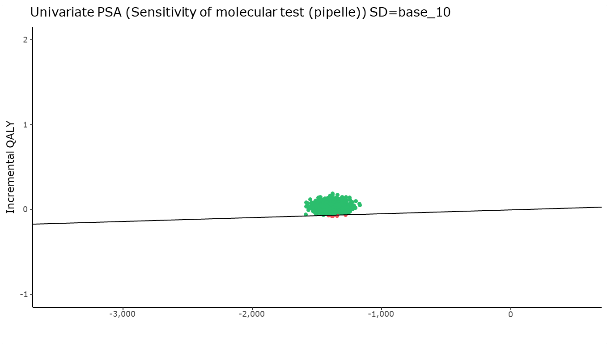** | **Figure S41. PSA for the specificity of the molecular test on pipelle.**  **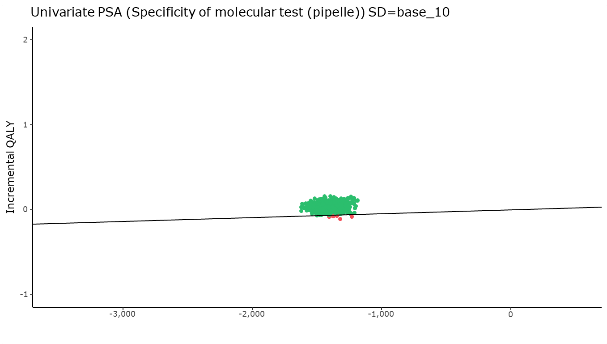** |
| --- | --- |
| **Figure S42. PSA for the sensitivity of pipelle.**  **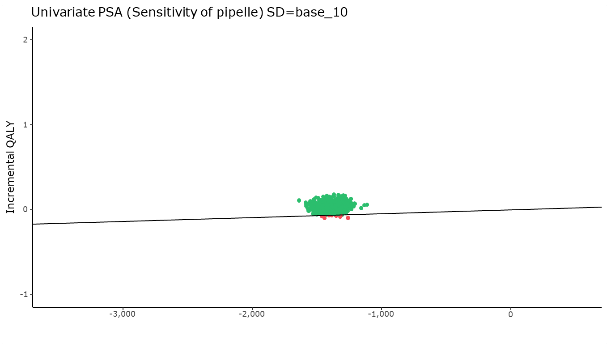** | **Figure S43. PSA for the specificity of pipelle.**  **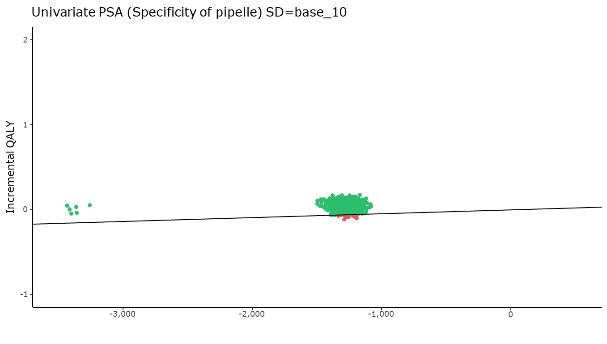** |
| **Figure S44. PSA for the sensitivity of TVU.**  **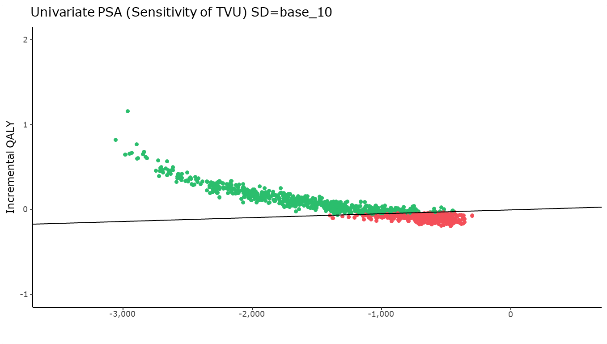** | **Figure S45. PSA for the specificity of TVU.**  **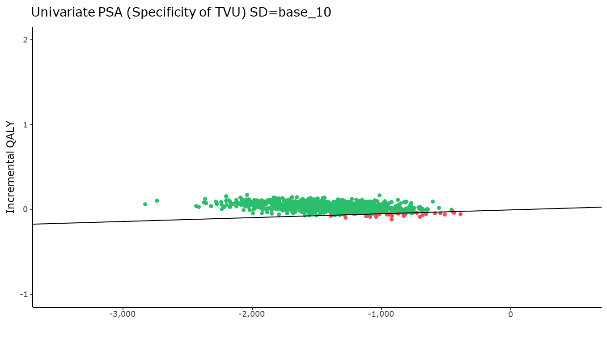** |
| **Figure S46. PSA for 5-year survival probability (stage I).**  **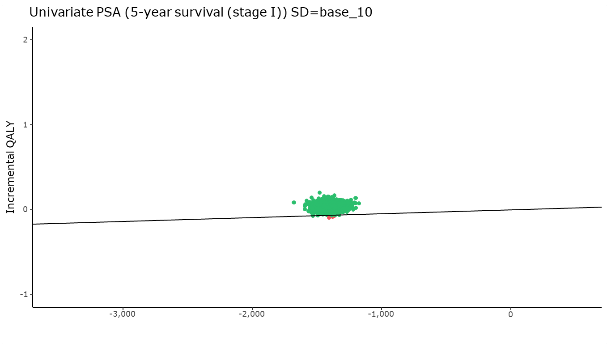** | **Figure S47. PSA for 5-year survival probability (stage II).**  **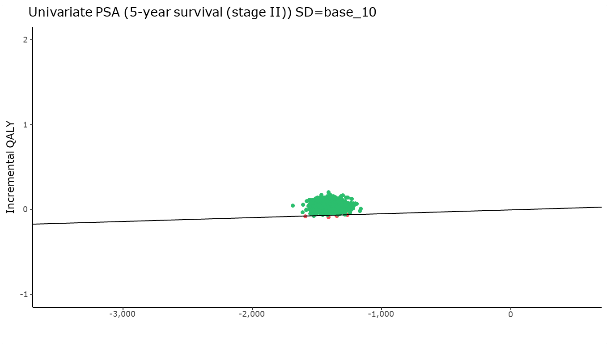** |

| **Figure S48. PSA for 5-year survival probability (stage III).**  **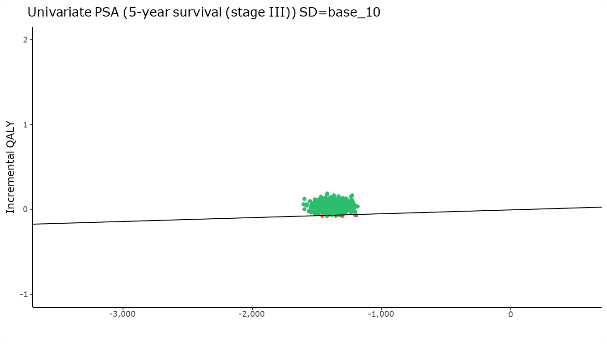** | **Figure S49. PSA for 5-year survival probability (stage IV).**  **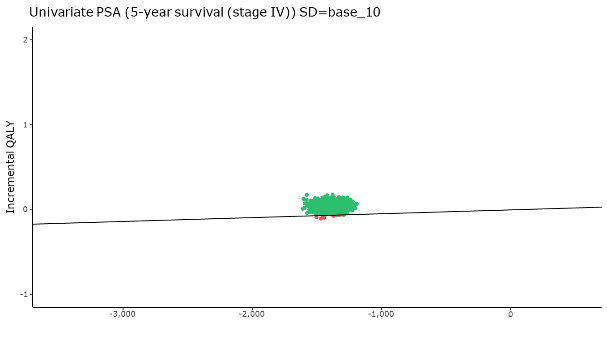** |
| --- | --- |
| **Figure S50. PSA for the probability of death from other causes.**  **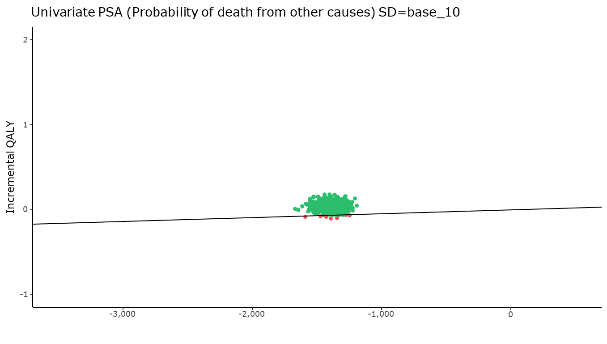** | **Figure S51. PSA for PMB utility.**  **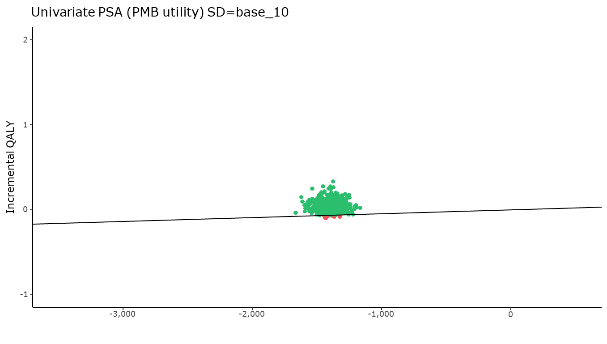** |
| **Figure S52. PSA for EC utility (stage I).**  **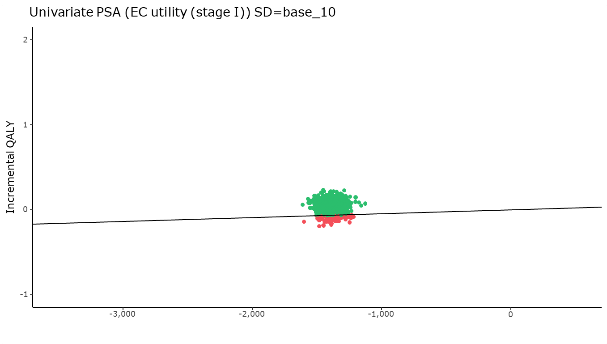** | **Figure S53. PSA for EC utility (stage II).**  **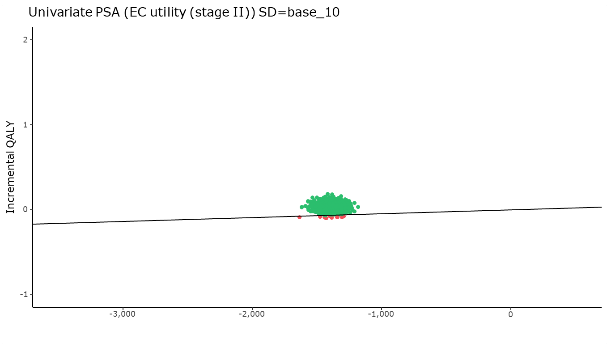** |
| **Figure S54. PSA for EC utility (stage III).**  **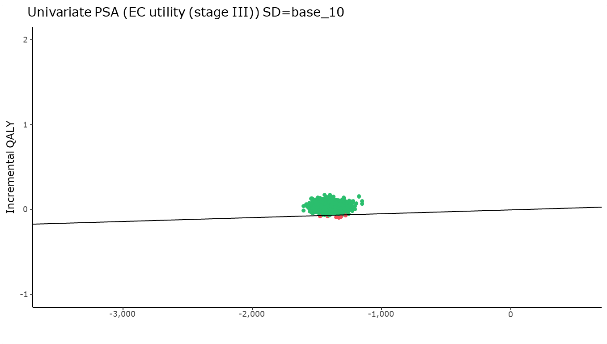** | **Figure S55. PSA for EC utility (stage IV).**  **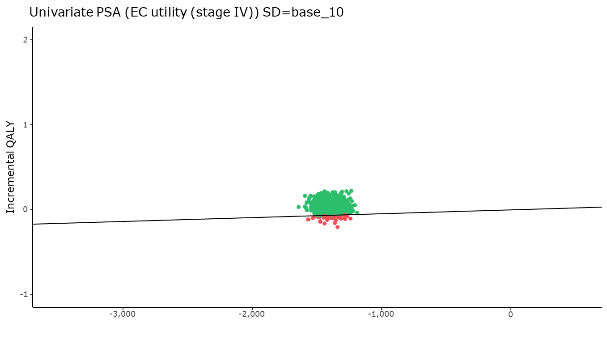** |

| **Figure S56. PSA for non-cancer hysterectomy utility.**  **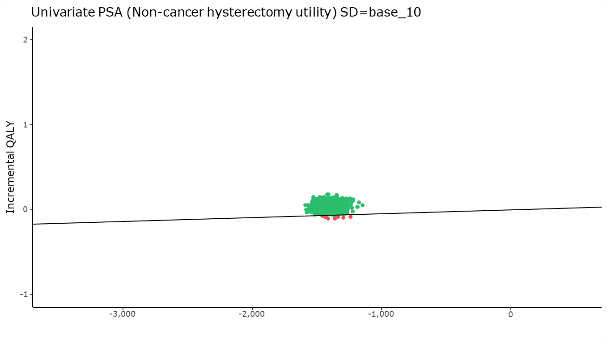** | **Figure S57. PSA for undetected EC utility.**  **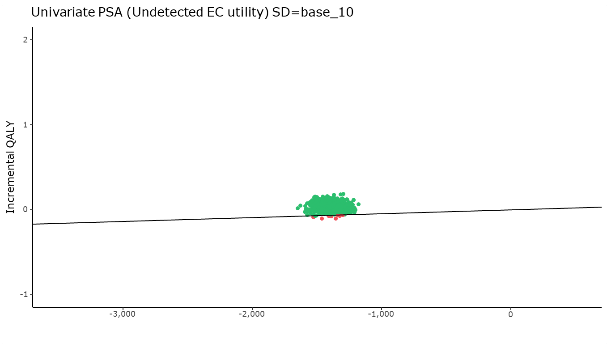** |
| --- | --- |

Ec, endometrial cancer; PMB, postmenopausal bleeding; pSA, probabilistic sensitivity analysis; TVU, transvaginal ultrasound.

### Figure S58. Multivariate probabilistic sensitivity analysis including bleeding persistence, sensitivity of TVU, specificity of TVU, utilities of EC stage I and stage IV.

**a) b)**

**c) d)**

*EC, endometrial cancer; ICER, incremental cost effectiveness ratio; TVU, transvaginal ultrasound; QALY, quality-adjusted life-years; WTP, willingness to pay.*

Figures A and C correspond to scatterplots displaying the incremental cost-effectiveness of the novel (molecular) strategy compared to the current strategy. The y-axis represents the incremental effectiveness, the x-axis represents the incremental cost and each dot represents a simulation. Different simulations are shown with colored dots: green dots represent simulations with a net health benefit (NHB) ≥0 which are cost-effective and the red dots represent those simulations with a NHB <0, which are not cost-effective. NHB is calculated as incremental effectiveness – incremental cost / WTP. Figures B and D are acceptability curves showing the percentage of cost-effective simulations (y-axis) of the current strategy (blue line) and the molecular strategy (orange line) for different WTP values (x-axis). Figures A and B use as standard deviation (SD) a tenth of the base case value (SD= base value/10) and figures C and D use a sixth of the base case value (SD=base value/6).

References

1. Husereau D, Drummond M, Augustovski F, de Bekker-Grob E, Briggs AH, Carswell C, et al. Consolidated Health Economic Evaluation Reporting Standards 2022 (CHEERS 2022) Statement: Updated Reporting Guidance for Health Economic Evaluations. Value in Health. 2022 Jan;25(1):3–9.

2. Ferlay J, Laversanne M, Ervik M, LAM F, Colombet M, Mery L, et al. Global Cancer Observatory: Cancer Today (Globocan 2020) [Internet]. Lyon, France: International Agency for Research on Cancer. 2020 [cited 2021 Mar 17]. Available from: https://gco.iarc.fr/today

3. Clarke MA, Long BJ, Del Mar Morillo A, Arbyn M, Bakkum-Gamez JN, Wentzensen N. Association of Endometrial Cancer Risk With Postmenopausal Bleeding in Women. JAMA Intern Med. 2018 Sep;178(9):1210–22.

4. INE. INE. Instituto Nacional de Estadística [Internet]. INE. [cited 2021 Mar 18]. Available from: https://www.ine.es/

5. Etzioni RD, Feuer EJ, Sullivan SD, Lin D, Hu C, Ramsey SD. On the use of survival analysis techniques to estimate medical care costs. Journal of health economics. 1999;365–80.

6. Jones E, Epstein D, García-Mochón L. A Procedure for Deriving Formulas to Convert Transition Rates to Probabilities for Multistate Markov Models. Med Decis Making. 2017 Oct;37(7):779–89.

7. Bhaskaran K, Douglas I, Forbes H, dos-Santos-Silva I, Leon DA, Smeeth L. Body-mass index and risk of 22 specific cancers: a population-based cohort study of 5·24 million UK adults. Lancet. 2014 Aug 30;384(9945):755–65.

8. Cancer of the Endometrium - Cancer Stat Facts [Internet]. SEER. [cited 2019 Sep 3]. Available from: https://seer.cancer.gov/statfacts/html/corp.html

9. Huijgens ANJ, Mertens HJMM. Factors predicting recurrent endometrial cancer. Facts Views Vis Obgyn. 2013;5(3):179–86.

10. Kwon JS, Sun CC, Peterson SK, White KG, Daniels MS, Boyd‐Rogers SG, et al. Cost‐effectiveness analysis of prevention strategies for gynecologic cancers in Lynch syndrome. Cancer. 2008 Jul 15;113(2):326–35.

11. Goldie SJ, Kohli M, Grima D, Weinstein MC, Wright TC, Bosch FX, et al. Projected clinical benefits and cost-effectiveness of a human papillomavirus 16/18 vaccine. J Natl Cancer Inst. 2004 Apr 21;96(8):604–15.

12. Department of Health. Official Journal of the Generalitat de Catalunya (DOGC). Resolució SLT/353/2013, de 13 de febrer, sobre la revisió de preus públics corresponents als serveis sanitaris que presta l’Institut Català de la Salut (Disposició derogada) | Departament de Salut | Legislació [Internet]. Cercador d’Informació i Documentació Oficials (CIDO) - Diputació de Barcelona. [cited 2020 Nov 2]. Available from: http://cido.diba.cat/legislacio/1669316/resolucio-slt3532013-de-13-de-febrer-sobre-la-revisio-de-preus-publics-corresponents-als-serveis-sanitaris-que-presta-linstitut-catala-de-la-salut-disposicio-derogada-departament-de-salut

13. Pytlak R. Limited Memory Quasi-Newton Algorithms. Book: Conjugate Gradient Algorithms in Nonconvex Optimization. [Internet]. 2009 [cited 2023 Mar 1]. Available from: https://link.springer.com/book/10.1007/978-3-540-85634-4

14. CatSalut-SCS (Guia). GeCofarma - Generando conocimiento sobre la prestación farmacéutica - Guía y recomendaciones para la realización y presentación de evaluaciones económicas y análisis de impacto presupuestario de medicamentos (GAEIP) del CatSalut [Internet]. 2014 [cited 2017 Mar 1]. Available from: http://www.gecofarma.catsalut.cat/esp/gestion_y_financiacion-evaluacion/guia_y_recomendaciones_para_la_realizacion_y_presentacion_de_evaluaciones_economicas_y_analisis_de_impacto_presupuestario_de_medicamentos_gaeip_del_catsalut.html
